# METTL14-mediated m6A epitranscriptomic modification contributes to chemotherapy-induced neuropathic pain by stabilizing GluN2A expression via IGF2BP2

**DOI:** 10.1172/JCI174847

**Published:** 2024-02-06

**Authors:** Weicheng Lu, Xiaohua Yang, Weiqiang Zhong, Guojun Chen, Xinqi Guo, Qingqing Ye, Yixin Xu, Zhenhua Qi, Yaqi Ye, Jingyun Zhang, Yuge Wang, Xintong Wang, Shu Wang, Qiyue Zhao, Weian Zeng, Junting Huang, Huijie Ma, Jingdun Xie

**Affiliations:** 1 Department of Anesthesiology, State Key Laboratory of Oncology in South China, Sun Yat-sen University Cancer Center, Guangzhou, Guangdong, China.; 2 Department of Physiology, Hebei Medical University, Shijiazhuang, Hebei, China.; 3 Department of Anatomy and Neurobiology, Zhongshan School of Medicine, Sun Yat-sen University, Guangzhou, Guangdong, China.

**Keywords:** Neuroscience, Epigenetics, Pain, Synapses

## Abstract

Epigenetics is a biological process that modifies and regulates gene expression, affects neuronal function, and contributes to pain. However, the mechanism by which epigenetics facilitates and maintains chronic pain is poorly understood. We aimed to determine whether *N*^6^-methyladenosine (m6A) specifically modified by methyltransferase-like 14 (METTL14) alters neuronal activity and governs pain by sensitizing the GluN2A subunit of the *N*-methyl-d-aspartate receptor (NMDAR) in the dorsal root ganglion (DRG) neurons in a model of chemotherapy-induced neuropathic pain (CINP). Using dot blotting, immunofluorescence, gain/loss-of-function, and behavioral assays, we found that m6A levels were upregulated in L4–L6 DRG neurons in CINP in a DBP/METTL14-dependent manner, which was also confirmed in human DRGs. Blocking METTL14 reduced m6A methylation and attenuated pain hypersensitivity. Mechanistically, METTL14-mediated m6A modification facilitated the synaptic plasticity of DRG neurons by enhancing the GluN2A subunit of NMDAR, and inhibiting METTL14 blocked this effect. In contrast, overexpression of METTL14 upregulated m6A modifications, enhanced presynaptic NMDAR activity in DRG neurons, and facilitated pain sensation. Our findings reveal a previously unrecognized mechanism of METTL14-mediated m6A modification in DRG neurons to maintain neuropathic pain. Targeting these molecules may provide a new strategy for pain treatment.

## Abstract 2

METTL14-mediated m6A modification contributes to chemotherapy-induced enhanced NMDAR-mediated glutamatergic input to spinal dorsal horn neurons and neuropathic pain by stabilizing GluN2A expression via IGF2BP2.

## Graphical abstract

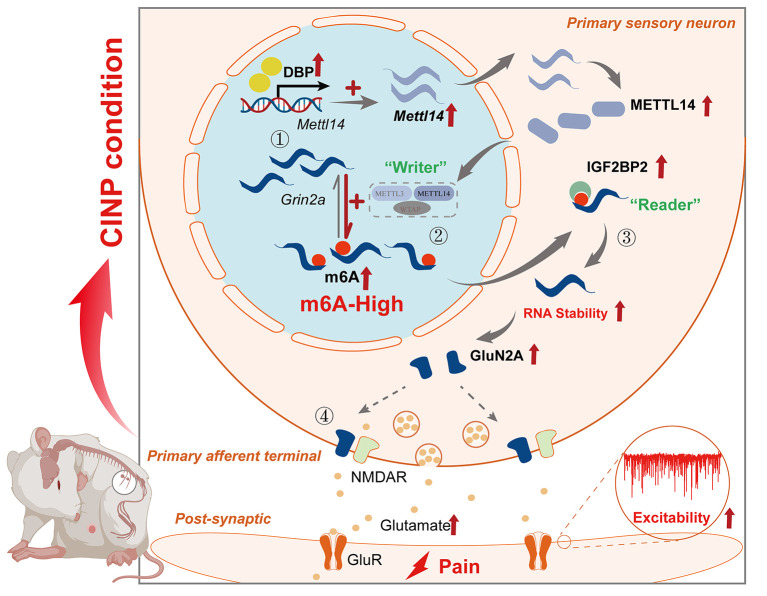

## Introduction

Chemotherapy-induced neuropathic pain (CINP) is a debilitating complication caused by damage to the peripheral nervous system by chemotherapeutic agents, including paclitaxel (1). CINP occurs in approximately 50% to 90% of cancer survivors receiving chemotherapy, resulting in therapy discontinuation or even disability (2). Currently, effective management of CINP remains limited. Therefore, there is an urgent need to elucidate the underlying mechanisms of CINP and identify novel drug targets.

Epigenetics refers to long-lasting heritable changes in gene expression that do not affect the DNA sequences (3). *N*^6^-methyladenosine (m6A) is the most ubiquitous post-transcriptional modification in eukaryotes (4). Characterized by a consensus motif (RRACH), m6A is critical for gene expression, as it regulates RNA localization, processing, translation, and decay. It preferentially affects the sequence of the mRNA stop codon and 3′-untranslated region (3′-UTR) (5–7) and is mainly mediated by RNA methyltransferase complexes, including methyltransferase-like 14 (METTL14), methyltransferase-like 3 (METTL3), and Wilms’ tumor 1–associating protein (WTAP) (8, 9). It can be reversibly removed by demethylases, including fat mass/obesity–associated protein (FTO) and alkylation repair homolog protein (ALKBH5) (10, 11). Furthermore, transcripts with m6A modifications can be recognized and interpreted by multiple m6A-binding proteins, such as insulin-like growth factor 2 mRNA–binding proteins (IGF2BPs) (12). m6A dysregulation is associated with various physiological and pathological processes, including pain. FTO in dorsal root ganglion (DRG) neurons contributes to nerve injury–associated pain hypersensitivity (13). METTL3 facilitates complete Freund’s adjuvant–induced (CFA-induced) inflammatory hyperalgesia by regulating TET1 expression in the spinal cord (14). Targeting novel epitranscriptomic mechanisms appears to be a promising strategy for the treatment of nociceptive disorders. However, the specific role of m6A modifications in CINP has not been explicitly defined.

*N*-methyl-d-aspartate receptors (NMDARs) are ligand-gated ion channels that are essential for synaptic plasticity (15). Conventionally, NMDARs are predominantly localized to the postsynaptic membrane. However, NMDARs are also expressed and act presynaptically, particularly at the central terminals of primary sensory neurons (16). Enhanced presynaptic NMDAR activity contributes to glutamate release from primary afferent terminals to spinal dorsal horn neurons under painful conditions (17, 18). Upregulation of GluN2A, a key subunit of NMDARs, is indispensable for increased presynaptic NMDAR activity in CINP (19). In this regard, blocking GluN2A-containing NMDARs at the spinal cord level diminishes paclitaxel-induced hyperalgesia (20, 21). Nevertheless, the mechanism underlying the chemotherapy-induced GluN2A upregulation in primary sensory neurons remains unclear.

In this study, we aimed to determine the role of m6A modification in paclitaxel-induced CINP and to uncover the epigenetics of NMDARs in nociception. We demonstrated that paclitaxel treatment increased the m6A abundance in the DRG in CINP. The RNA methyltransferase METTL14 was highly expressed and contributed to CINP. GluN2A, but not GluN1 or GluN2B, in the DRG was a downstream target of METTL14. Mechanistically, METTL14-mediated m6A modification facilitated CINP by stabilizing *Grin2a* (coding for GluN2A) in an IGF2BP2-dependent manner, contributing to presynaptic NMDAR hyperactivity at the spinal dorsal horn level. Furthermore, the D-box–binding PAR BZIP transcription factor (DBP) contributed to the paclitaxel-induced increase in METTL14 expression by activating *Mettl14* transcription in the DRG. Together, our findings provide new insight into the molecular pathogenesis of CINP.

## Results

### Paclitaxel treatment increases m6A abundance and METTL14 expression in rat and human DRG.

**Figure 1:**
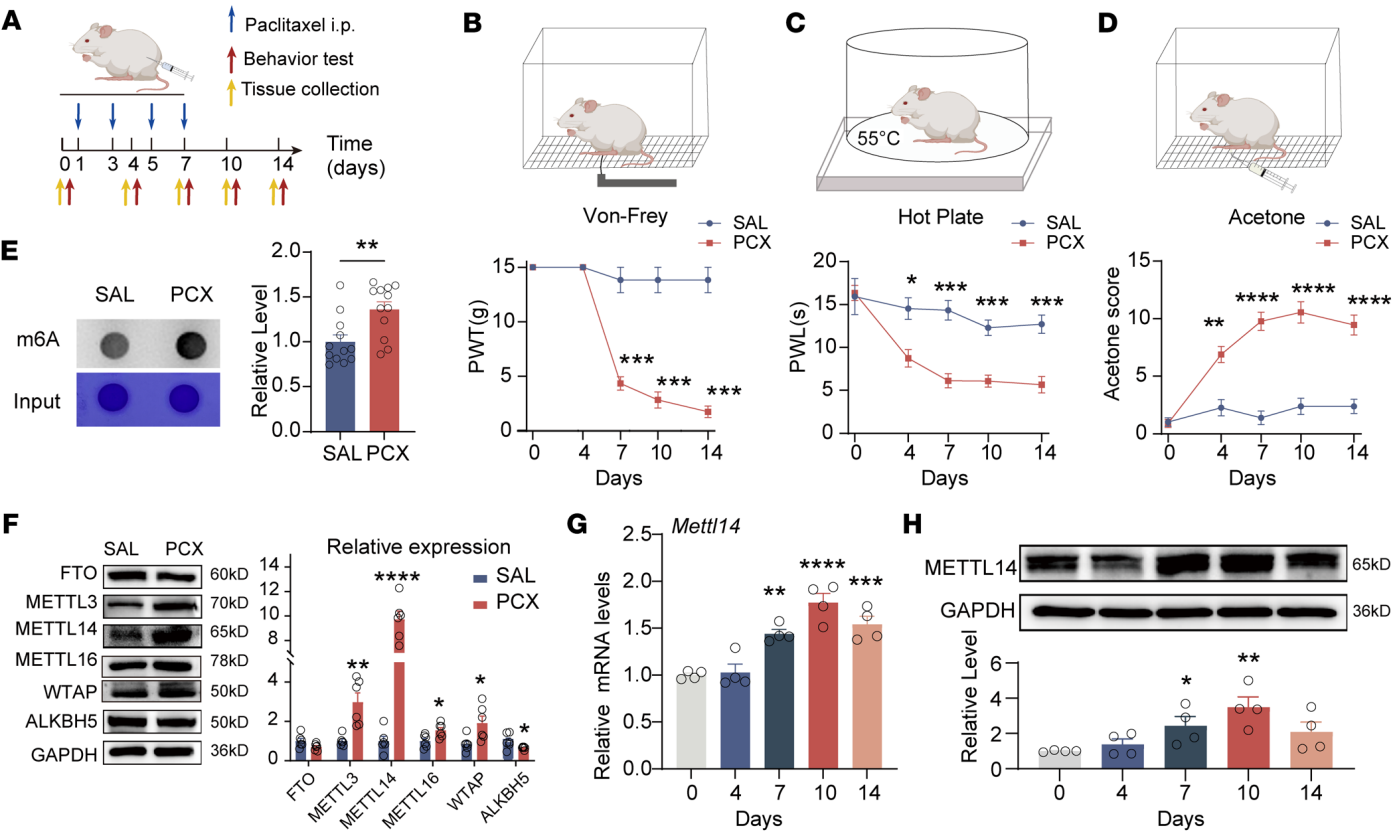
Paclitaxel treatment increases m6A levels and METTL14 expression in the DRG. (**A**) Timeline of paclitaxel treatment, behavioral assessment, and tissue collection in rats. (**B**–**D**) Paclitaxel induced long-lasting notable mechanical allodynia, thermal hyperalgesia, and cold pain in rats (at least 6 rats per group, 2-way ANOVA followed by Šidák’s post hoc test). PWT, paw withdrawal threshold; PWL, paw withdrawal latency. (**E**) Representative dot blot images and quantification showing the m6A level in the bilateral L4–L6 DRGs in rats 10 days after treatment with saline or paclitaxel (PCX) (*n* = 12 rats per group, Student’s *t* test). (**F**) Representative immunoblot images and quantification show the protein levels of FTO, METTL3, METTL14, METTL16, WTAP, and ALKBH5 in the bilateral L4–L6 DRGs in rats 10 days after treatment with saline or PCX (*n* = 6 rats per group, Student’s *t* test). (**G** and **H**) Quantification and representative immunoblot images show the mRNA and protein levels of METTL14 in the bilateral L4–L6 DRGs in rats 0–14 days after treatment with PCX (*n* = 4 rats per group, 1-way ANOVA followed by Dunnett’s post hoc test for mRNA and least significant difference post hoc test for protein). *P < 0.05, **P < 0.01, ***P < 0.001, ****P < 0.0001.

Following paclitaxel administration, the rats developed long-lasting exaggerated pain behaviors, as indicated by a marked and sustained decrease in the paw withdrawal threshold, a decrease in paw withdrawal latency, and an increase in acetone response scores (Figure 1, A–D). To systematically determine m6A abundance and associated enzyme alterations in the DRG of CINP, we conducted m6A dot blot and Western blot assays. A dot blot assay revealed that the total m6A levels in the DRG were notably upregulated by paclitaxel treatment (Figure 1E). Furthermore, the expression levels of the methyltransferases METTL3, METTL14, METTL16, and WTAP increased whereas the expression levels of the demethylase ALKBH5 decreased in the DRG after paclitaxel treatment (Figure 1F). As the elevation in METTL14 expression in the DRG caused by paclitaxel was most evident, we chose METTL14 for further studies.

**Figure 2:**
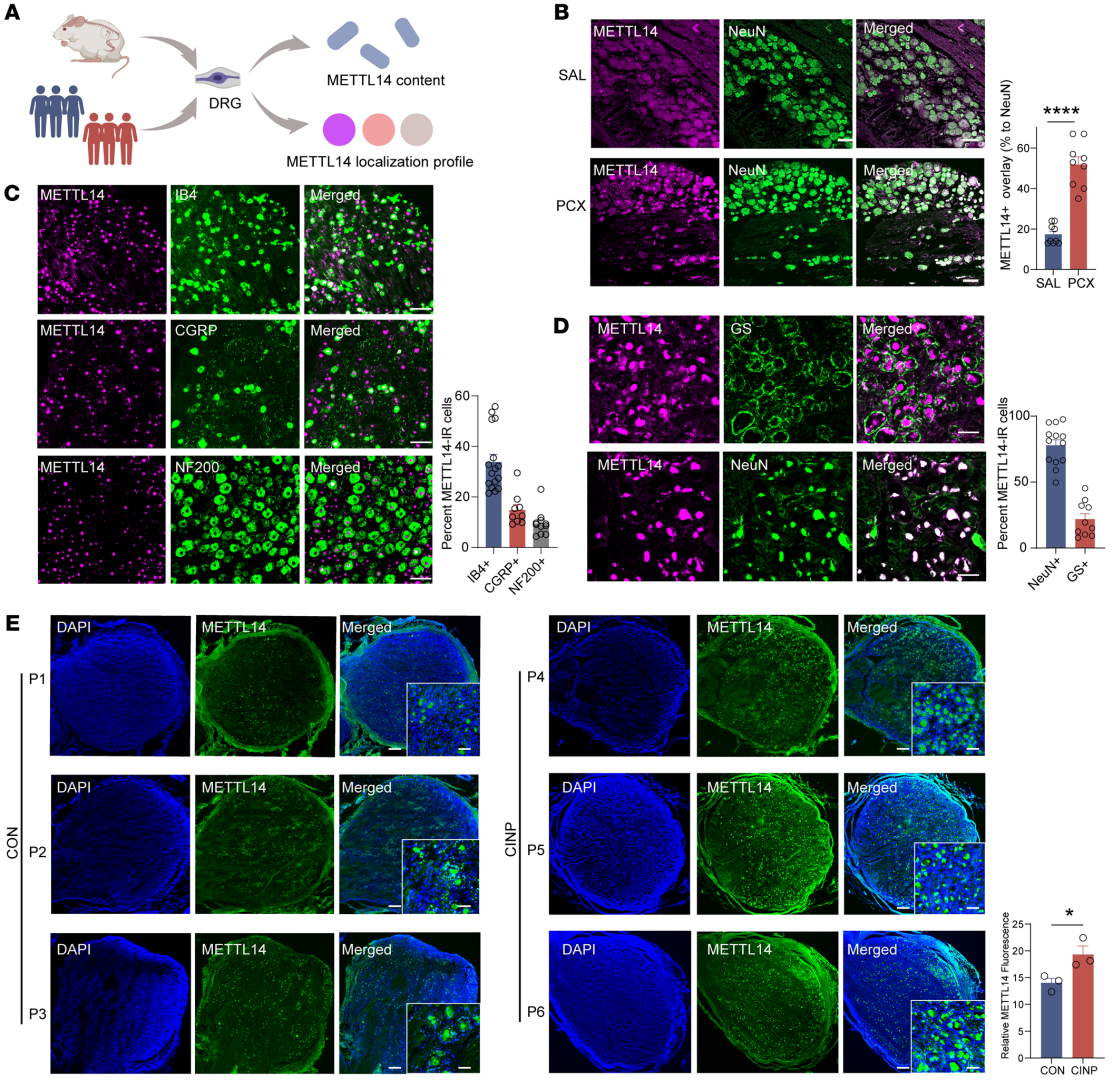
Characterization of METTL14 expression profile in the rat and human DRG. (**A**) Experimental paradigm for METTL14 expression profile analysis. (**B**) Left: Representative immunofluorescence images show colabeling of METTL14 and NeuN in the rat DRG 10 days after treatment with saline or PCX. Right: The percentage of DRG neurons colabeled by METTL14 and NeuN in the L4 and L5 DRGs 10 days after treatment with saline or PCX (*n* = 9 DRGs from 3 rats per group, Student’s *t* test). Scale bars: 100 μm. (**C**) Representative double immunofluorescence staining and quantification showing the colocalization of METTL14 with IB4, CGRP, and NF200 in rat DRG (at least 9 DRG slices from 4 rats per group). Scale bars: 100 μm. (**D**) Representative double immunofluorescence staining and quantification showing the colocalization of METTL14 with NeuN and glutamine synthetase (GS) in human DRG (*n* = 10 DRG slices per group). Scale bars: 50 μm. (**E**) Representative immunofluorescence staining and quantification showing colabeling and alteration of METTL14 and DAPI in patients (*n* = 3 patients per group, Student’s *t* test). Scale bars: 100 μm. Data are shown as the mean ± SEM. *P < 0.05, ****P < 0.0001.

We investigated *Mettl14* mRNA and protein levels using real-time quantitative PCR (qPCR) and Western blotting in rat DRG at different time points after paclitaxel treatment. A notable increase was observed from day 7, reaching the highest level on day 10 after paclitaxel treatment (Figure 1, G and H). Next, we characterized the METTL14 expression profile in DRG (Figure 2A). Double immunolabeling confirmed that METTL14 was expressed predominantly in DRG neurons (NeuN-positive cells) (Figure 2B), but less in satellite glial cells (GFAP-positive cells) and macrophages (IBA1-positive cells) (Supplemental Figure 1, A and B; supplemental material available online with this article; https://doi.org/10.1172/JCI174847DS1). The colocalization of METTL14 in DRG neurons consistently increased after paclitaxel treatment as previously indicated (Figure 2B). Moreover, double-labeling immunofluorescence showed that METTL14-positive cells were predominantly neurons positive for IB4 (a marker for DRG non-peptidergic neurons; 33.87% ± 3.007%) and calcitonin gene–related peptide (CGRP) (a marker for small DRG peptidergic neurons; 14.9% ± 2.123%), but fewer were neurons positive for neurofilament-200 (NF200) (a marker for medium/large DRG neurons with myelinated Aβ fibers; 9.587% ± 1.523%) (Figure 2C). Further results consistently indicated that human DRG METTL14 was also expressed mostly in neurons (NeuN-positive cells, 77.87% ± 4.198%) but less in glial cells (glutamine synthetase–positive [GS-positive] cells, 21.91% ± 4.188%) (Figure 2D). Increased METTL14 expression was confirmed in the DRG tissues of clinical patients with chemotherapeutic agents, but not in those without treatment, and was mainly located in the nucleus (costained with DAPI dihydrochloride, a nuclear staining marker) (Figure 2E). Taken together, these results indicate that paclitaxel treatment increases m6A abundance and METTL14 expression in rat and human DRG.

### Paclitaxel increases m6A levels in the DRG via METTL14.

**Figure 3:**
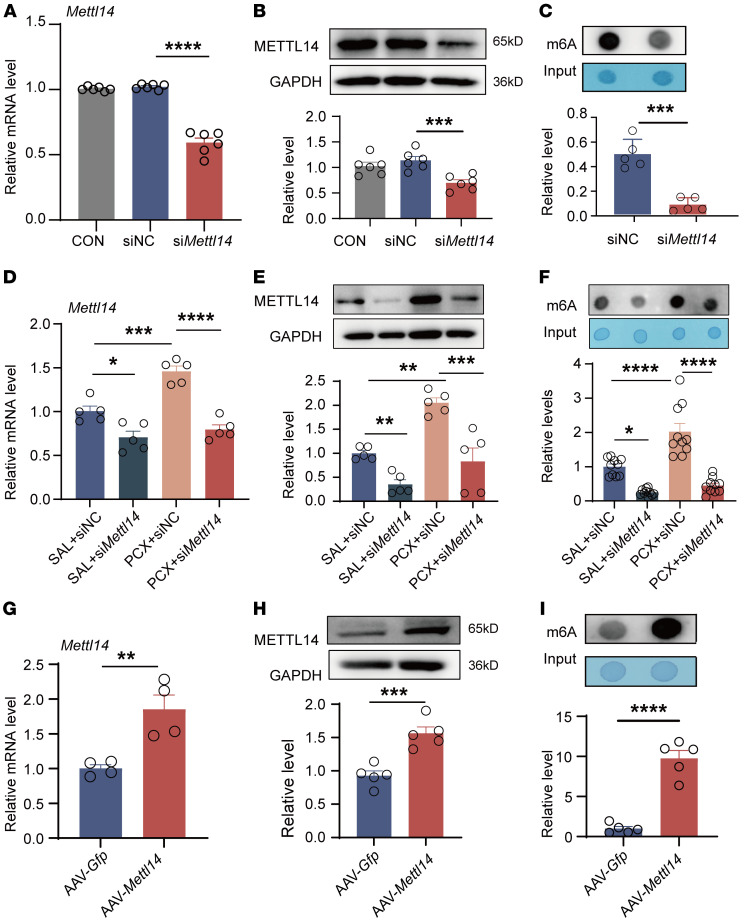
Paclitaxel treatment increases the global m6A level in the DRG via METTL14. (**A** and **B**) *Mettl14* mRNA (**A**) and protein (**B**) expression in the bilateral L4–L6 DRGs in naive rats after si*Mettl14* or scrambled siRNA intrathecal injection (*n* = 6 rats per group, 1-way ANOVA followed by Tukey’s post hoc test). (**C**) RNA m6A level in the bilateral L4–L6 DRGs in naive rats after si*Mettl14* or scrambled siRNA treatment (*n* = 5 rats per group, Student’s *t* test). (**D** and **E**) *Mettl14* mRNA (**D**) and protein (**E**) expression in the bilateral L4–L6 DRGs in saline- or PCX-pretreated rats after si*Mettl14* intrathecal injection (*n* = 5 rats per group, 1-way ANOVA followed by Tukey’s post hoc test). (**F**) RNA m6A level in the bilateral L4–L6 DRGs in saline- or PCX-pretreated rats after si*Mettl14* or scrambled siRNA treatment (*n* = 10 rats per group, 1-way ANOVA followed by Tukey’s post hoc test). (**G** and **H**) *Mettl14* mRNA (**G**) and protein (**H**) expression in the bilateral L4–L6 DRGs in naive rats after AAV-*Mettl14* or AAV-*Gfp* intrathecal injection (at least 4 rats per group, Student’s *t* test). (**I**) RNA m6A level in the bilateral L4–L6 DRGs in naive rats after AAV-*Mettl14* or AAV-*Gfp* injection (*n* = 5 rats per group, Student’s *t* test). Data are shown as the mean ± SEM. *P < 0.05, **P < 0.01, ***P < 0.001, ****P < 0.0001.

Next, we investigated whether METTL14 regulates m6A levels in CINP DRG. Initially, we screened the siRNA sequences in PC12 cells for METTL14 ablation (Supplemental Figure 2A). Among the screened sequences, siRNA-*Mettl14*-03 (named si*Mettl14* hereafter) resulted in the most effective knockdown of *Mettl14* mRNA. *Mettl14* mRNA and protein expression in the DRG was decreased by approximately 50% following si*Mettl14* intrathecal injection in naive rats (Figure 3, A and B), compared with that of scrambled siRNA. Furthermore, treatment with si*Mettl14* in naive rats markedly decreased m6A levels in the L4–L6 DRGs (Figure 3C).

We determined the role of METTL14 in regulating paclitaxel-induced m6A expression. METTL14 knockdown using a si*Mettl14* reduced the paclitaxel-induced increase in *Mettl14* mRNA and protein levels in the DRG (Figure 3, D and E). Consistently, si*Mettl14* reduced m6A levels in the DRG potentiated by paclitaxel treatment (Figure 3F).

We also determined whether METTL14 overexpression affected m6A levels in the DRG. AAV9 expressing full-length *Mettl14* (AAV-*Mettl14*) was injected intrathecally to overexpress METTL14 in the DRG of naive rats. The results showed that AAV-*Mettl14* induced high *Mettl14* mRNA and protein expression in the rat DRG (Figure 3, G and H), in which obvious FLAG-positive signal in neurons and upregulation of METTL14 immunofluorescence signaling indicated the high transfection efficiency of AAV-*Mettl14* carrying FLAG label (Supplemental Figure 2, B and C). A dot blot assay showed that METTL14 overexpression in naive rats caused an increase in m6A levels in the DRG (Figure 3I). Collectively, our results suggest that METTL14 mediates paclitaxel-induced m6A upregulation in the DRG.

### METTL14 contributes to pain hypersensitivity induced by paclitaxel treatment.

**Figure 4:**
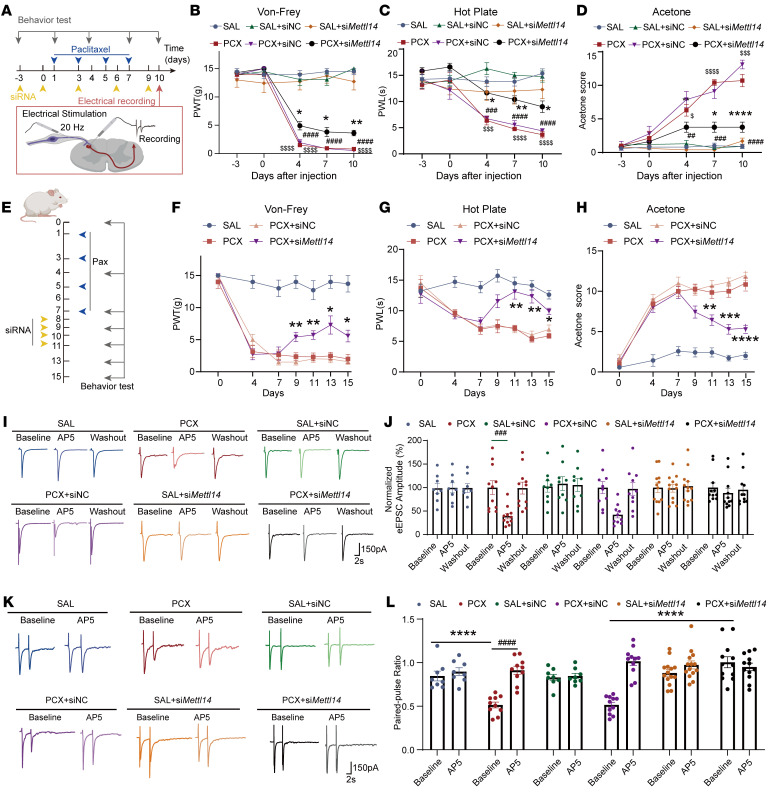
Paclitaxel treatment induces tactile and thermal hypersensitivity and potentiates glutamate release from primary afferent nerve terminals via METTL14. (**A**) Illustration of the experimental design. (**B**–**D**) Mechanical allodynia, thermal hyperalgesia, and cold allodynia of saline- or PCX-treated rats after si*Mettl14* or scrambled siRNA treatment measured by the von Frey test (**B**), hot plate test (**C**), and acetone test (**D**), respectively (at least 7 rats per group; *PCX+si*Mettl14-* vs. PCX+siNC-treated rats, ^#^PCX+siNC- vs. SAL+siNC-treated rats, ^$^PCX- vs. SAL-treated rats). siNC, negative control RNA. (**E**) Schematic of the METTL14 therapeutic role evaluation. (**F**–**H**) Mechanical allodynia, thermal hyperalgesia, and cold allodynia were measured following PCX treatment and siRNA injection (7 rats per group, 2-way ANOVA followed by Tukey’s post hoc test; *PCX+si*Mettl14* vs. PCX+siNC). (**I** and **J**) Representative recording traces and quantification show the baseline control and the effect of bath application of AP5 on monosynaptic EPSCs of a lamina II neuron evoked from the dorsal root from saline- or PCX-pretreated rats after si*Mettl14* or scrambled siRNA treatment. (**K** and **L**) Representative recording traces and quantification show the baseline control and the effect of bath application of AP5 on the EPSCs evoked by a pair of pulses from saline- or PCX-pretreated rats after si*Mettl14* or scrambled siRNA treatment (at least 3 rats, 8 neurons per group; *compared with baseline between each group, ^#^compared with the respective baseline). *P < 0.05, **P < 0. 01, ***P < 0.001, ****P < 0. 0001; ^##^*P* < 0.01, ^###^*P* < 0.001, ^####^*P* < 0.0001; ^$^*P* < 0.01, ^$$$^*P* < 0.001, ^$$$$^*P* < 0.0001. Two-way ANOVA followed by Tukey’s post hoc test.

To determine the role of METTL14 in the development of CINP, we intrathecally injected si*Mettl14* or scrambled control siRNA into rats treated with saline or paclitaxel (Figure 4A). Results showed that si*Mettl14* intrathecal injection notably attenuated paclitaxel-induced pain behaviors, including mechanical hypersensitivity (Figure 4B), thermal hyperalgesia (Figure 4C), and cold allodynia (Figure 4D). We also measured pain thresholds in female rats treated with si*Mettl14* and found that *Mettl14* siRNA alleviated paclitaxel-induced mechanical, thermal, and cold allodynia in female rats, indicating no potential sex-dependent effects of siRNA in CINP (Supplemental Figure 3, A–C). Additionally, to determine whether METTL14 manipulation can serve as a potential treatment strategy for CINP, we applied si*Mettl14* after day 7, when the neuropathic pain was stably established. We found that siRNA administration for 4 consecutive days from day 8 attenuated paclitaxel-induced nociceptive behaviors (Figure 4, E–H), suggesting that targeting METTL14 is a promising treatment approach for CINP. These data suggest that METTL14 plays a crucial role in the development of CINP.

### METTL14 mediates paclitaxel treatment–induced presynaptic NMDAR hyperactivity in the spinal cord.

We then determined whether METTL14 contributes to paclitaxel-induced presynaptic NMDAR hyperactivity at the spinal cord level. Double-labeling immunofluorescence was conducted and confirmed that METTL14 was also predominantly colocalized with NeuN-positive cells in lamina I–III of spinal dorsal horns (81.58% ± 2.82%) (Supplemental Figure 4A). To elucidate the potential role of METTL14 at the primary sensory neuron terminal and spinal cord levels, we performed whole-cell patch-clamp recordings of spinal cord slices with dorsal root input in lamina II neurons, which preferentially receive nociceptive input from primary afferent nerves (22). Whole-cell recordings showed that the baseline frequency, but not the amplitude, of miniature excitatory postsynaptic currents (mEPSCs) in dorsal horn lamina II neurons was considerably higher in paclitaxel-treated rats than in control rats (Supplemental Figure 4, B–D). Further incubation with 2-amino-5-phosphonopentanoic acid (AP5; a specific NMDAR antagonist) restored the elevated mEPSC frequency in paclitaxel-treated rats (Supplemental Figure 4, B and C). Importantly, for the first time to our knowledge, we found that si*Mettl14*, but not control siRNA, largely normalized the elevated baseline frequency of mEPSCs in dorsal horn neurons caused by paclitaxel administration (Supplemental Figure 4, B and C).

To precisely elucidate the role of METTL14 in paclitaxel-induced NMDAR hyperactivity at primary afferent central terminals, we recorded monosynaptic EPSCs evoked by electrical dorsal root stimulation (eEPSCs) of dorsal horn neurons at day 10 (Figure 4A), which are widely used to evaluate presynaptic function (21, 23, 24). The results showed that the AP5 bath application normalized the elevated amplitude of evoked EPSCs in paclitaxel-treated rats but had no such effect in vehicle-treated rats (Figure 4, I and J). Intrathecal treatment with si*Mettl14*, but not with control siRNA, normalized the paclitaxel-induced increase in the amplitude of evoked EPSCs in spinal dorsal horn neurons (Figure 4, K and L). Additionally, to better determine the presynaptic action, we examined the paired-pulse ratio (PPR; the ratio of the second synaptic response amplitude to the first synaptic response, a common approach to studying the presynaptic strength of synaptic transmission; refs. 25, 26) of eEPSCs from rats treated with si*Mettl14* or control siRNA. si*Mettl14*, but not control siRNA, restored the PPR of paclitaxel-treated eEPSCs (Figure 4, K and L). Collectively, these results demonstrate that METTL14 is involved in paclitaxel-induced NMDAR hyperactivity at primary afferent terminal and spinal cord levels.

### METTL14 overexpression causes pain hypersensitivity and presynaptic NMDAR hyperactivity in the spinal cord.

**Figure 5:**
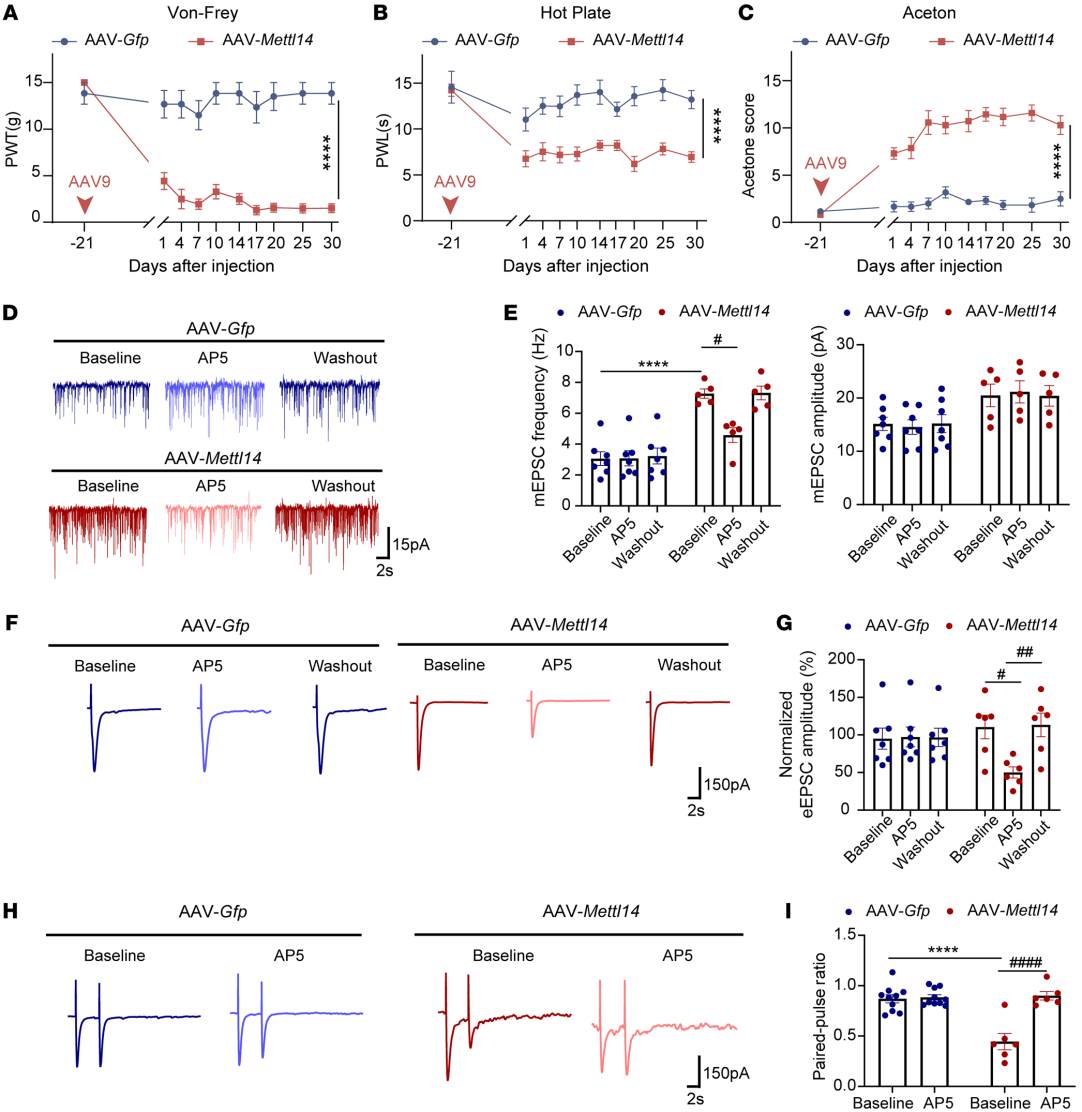
METTL14 overexpression induces mechanical, thermal, and cold hypersensitivity and presynaptic NMDAR hyperactivity in the spinal cord. (**A**–**C**) AAV-mediated overexpression of METTL14 induced long-lasting exaggerated mechanical allodynia, thermal hyperalgesia, and cold allodynia (at least 6 rats per group). (**D** and **E**) Representative recording traces and quantification show the effect of bath application of AP5 on the frequency and amplitude of mEPSCs of lamina II neurons from naive rats after AAV-*Mettl14* or AAV-*Gfp* injection (at least 5 neurons per group). (**F**–**I**) Representative recording traces and bar plots show the effect of bath application of AP5 on the normalized amplitude of evoked mEPSCs and PPR of lamina II neurons evoked from the dorsal root in naive rats after AAV-*Mettl14* or AAV-*Gfp* injection (at least 5 neurons per group were used; ^#^compared with the respective AP5-treated group, *compared with the baseline in the vehicle-treated group). Data are shown as the mean ± SEM. ****P < 0.0001; ^#^*P* < 0.05, ^##^*P* < 0.01, ^####^*P* < 0.0001. Two-way ANOVA followed by Tukey’s post hoc test.

We then determined whether increased METTL14 expression induces pain hypersensitivity. To this end, we intrathecally injected AAV-*Mettl14* into naive rats. AAV-*Mettl14*, but not the vehicle control, induced notable mechanical allodynia, thermal hyperalgesia, and cold allodynia during the entire monitoring period (Figure 5, A–C). To further elucidate whether increased METTL14 expression potentiates presynaptic NMDAR activity, we used a whole-cell patch clamp to measure the associated neuronal activity. As expected, AAV-induced METTL14 overexpression markedly increased the baseline frequency, but not the amplitude, of mEPSCs in spinal dorsal horn neurons (Figure 5, D and E). AP5 application mitigated the increase in the frequency induced by METTL14 overexpression (Figure 5, D and E). Most importantly, AP5 rapidly decreased the normalized amplitude of EPSCs evoked from the dorsal root, which was increased by METTL14 overexpression (Figure 5, F and G). Similarly, AP5 application reversed the PPR of the evoked EPSCs altered by METTL14 overexpression (Figure 5, H and I). These data suggest that METTL14 upregulation induces pain hypersensitivity and NMDAR hyperactivity in primary afferent terminals.

### METTL14 induces pain hypersensitivity and presynaptic NMDAR hyperactivity at the spinal cord level by targeting Grin2a.

**Figure 6:**
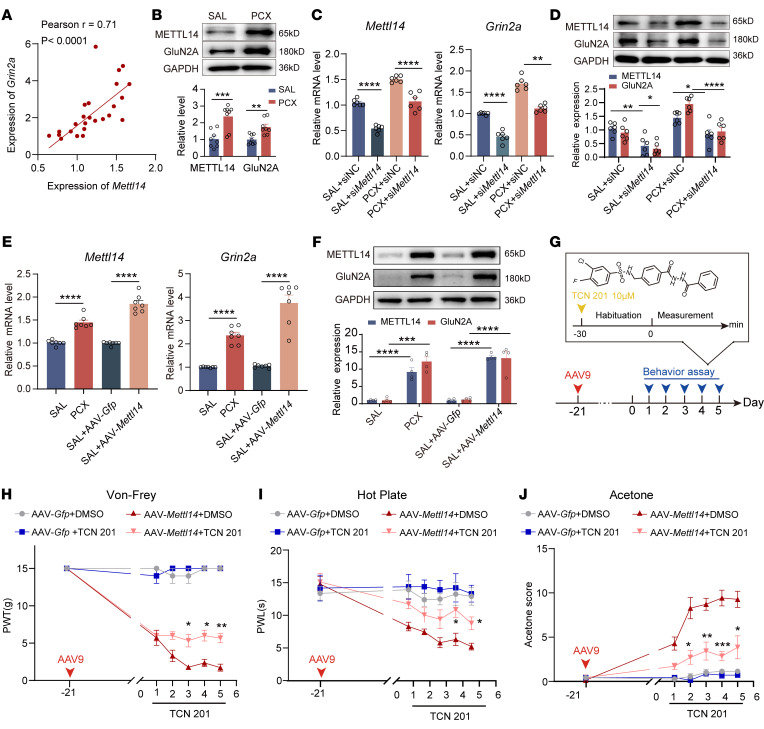
Paclitaxel treatment or METTL14 overexpression increases GluN2A expression and pain hypersensitivity. (**A**) Correlations between *Grin2a* and *Mettl14* mRNA expression were calculated using Pearson’s correlation. (**B**) METTL14 protein expression in the bilateral L4–L6 DRGs in saline- or PCX-pretreated rats (*n* = 8 rats per group, Student’s *t* test). (**C** and **D**) *Mettl14* and *Grin2a* mRNA (**C**) and protein (**D**) expression in the bilateral L4–L6 DRGs in saline- or PCX-pretreated rats after si*Mettl14* or scrambled siRNA intrathecal injection (*n* = 6 rats per group, 1-way ANOVA followed by Tukey’s post hoc test). (**E** and **F**) *Mettl14* and *Grin2a* mRNA (**E**) and protein (**F**) expression in the bilateral L4–L6 DRGs in saline- or PCX-pretreated rats after AAV-*Mettl14* or AAV-*Gfp* intrathecal injection (*n* = 7 rats per group for mRNA, 4 rats per group for protein, 1-way ANOVA followed by Tukey’s post hoc test). (**G**) Illustration of the TCN 201 application in experimental design. (**H**–**J**) Mechanical allodynia (**H**), thermal hyperalgesia (**I**), and cold allodynia (**J**) of naive rats after intrathecal injection of AAV-*Mettl14* or AAV-*Gfp* combined with TCN 201 or DMSO treatment (at least 7 rats per group; *AAV-*Mettl14*+TC201- vs. AAV-*Mettl14*+DMSO-treated rats; 2-way ANOVA followed by Tukey’s post hoc test). Data are shown as the mean ± SEM. *P < 0.05, **P < 0.01, ***P < 0.001, ****P < 0.0001.

We previously found that paclitaxel induces the activation of GluN2A-containing NMDARs at primary afferent central terminals (21). As METTL14 overexpression similarly increased presynaptic NMDAR activity, we determined whether *Grin2a* is a target of METTL14 in the DRG. Paclitaxel treatment observably increased the mRNA levels of *Mettl14*, *Grin2a*, and *Grin2b* in the DRG (Supplemental Figure 5A). Pearson’s correlation analysis showed that increased *Grin2a* and *Grin2b* expression, but not *Grin1* expression, was positively correlated with *Mettl14* upregulation following paclitaxel treatment, with the highest correlation observed for *Grin2a* (*r* = 0.71, *P* < 0.0001) (Figure 6A and Supplemental Figure 5B). Western blotting also confirmed the increased levels of METTL14 and GluN2A in the DRG (Figure 6B). The expression of GluN1 and GluN2B was also explored (Supplemental Figure 5C).

Moreover, intrathecal treatment with si*Mettl14*, but not control siRNA, attenuated paclitaxel-induced upregulation of *Grin2a* and *Grin2b*, but not *Grin1*, in the rat DRG (Figure 6, C and D, and Supplemental Figure 5D). Conversely, AAV9-mediated *Mettl14* overexpression markedly increased the expression of *Grin2a* and *Grin2b*, but not *Grin1*, in the DRG, with *Grin2a* exhibiting the most dramatic change (Figure 6, E and F, and Supplemental Figure 5E). These results suggest that *Grin2a*, but not *Grin1* or *Grin2b*, is a potential downstream target of METTL14 in the DRG.

Next, we determined the role of GluN2A in METTL14-induced pain hypersensitivity and presynaptic NMDAR hyperactivity. Intrathecal injection of TCN 201, a specific antagonist of GluN2A-containing NMDARs (27), observably reversed mechanical allodynia, thermal hyperalgesia, and cold allodynia induced by METTL14 overexpression in naive rats (Figure 6, G–J).

**Figure 7:**
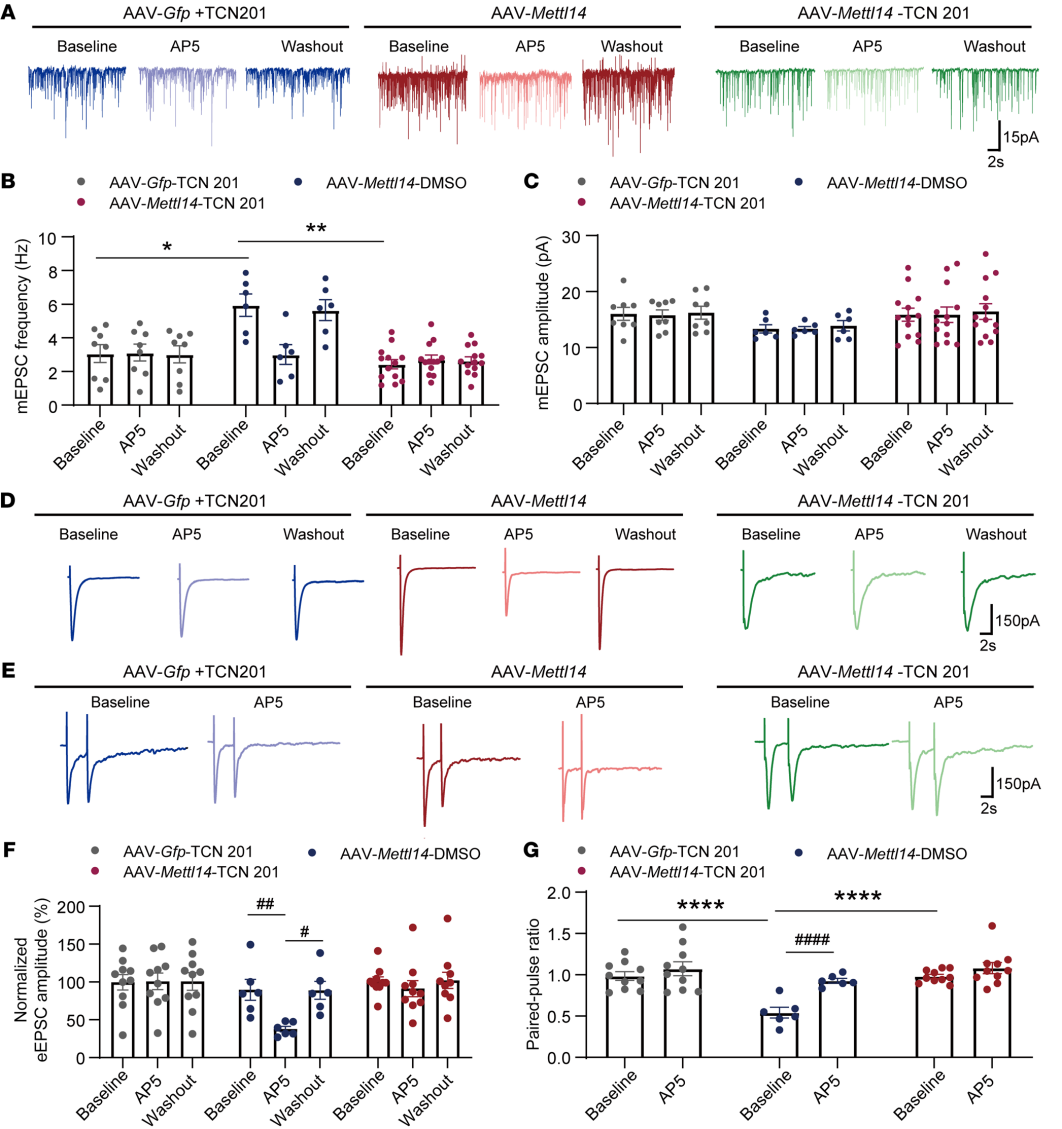
METTL14 overexpression increases GluN2A-mediated presynaptic NMDAR hyperactivity in the spinal cord. (**A**–**C**) Representative recording traces and quantification showing the effect of bath application of AP5 on the frequency and amplitude of mEPSCs of lamina II neurons from naive rats after AAV-*Mettl14* or AAV-*Gfp* pretreatment combined with intrathecal injection of TCN 201 (at least 6 neurons from 3 rats per group; 2-way ANOVA followed by Tukey’s post hoc test). (**D**) Representative recording traces show the effect of bath application of AP5 on the normalized amplitude of evoked mEPSCs of a lamina II neuron evoked from the dorsal root in naive rats after AAV-*Mettl14* or AAV-*Gfp* pretreatment combined with intrathecal injection of TCN 201. (**E**) Representative recording traces show the effect of bath application of AP5 on EPSCs evoked by a pair of pulses of a lamina II neuron evoked from the dorsal root in naive rats after AAV-*Mettl14* or AAV-*Gfp* pretreatment combined with intrathecal injection of TCN 201. (**F** and **G**) Bar plots show the change in the normalized amplitude of evoked mEPSCs (**F**) and the PPR of evoked EPSCs (**G**) during baseline control and bath application of AP5 in naive rats after AAV-*Mettl14* or AAV-*Gfp* pretreatment combined with intrathecal injection of TCN 201 (at least 3 rats, 6 neurons per group; ^#^compared with the respective baseline control, *compared with the baseline in the vehicle-treated group; 1-way ANOVA followed by Tukey’s post hoc test was performed in each group for the normalized amplitude of evoked mEPSCs, 2-way ANOVA followed by Šidák’s post hoc test between groups for PPR). Data are shown as the mean ± SEM. *P < 0.05, **P < 0.01, ****P < 0.0001; ^#^*P* < 0.05, ^##^*P* < 0.01, ^####^*P* < 0.0001.

Moreover, whole-cell recordings showed that GluN2A inhibition with TCN 201 reversed the high baseline frequency, but not the amplitude, of mEPSCs in dorsal horn neurons induced by METTL14 overexpression (Figure 7, A–C). In contrast, no differences were detected after bath application of AP5, indicating increased activity of GluN2A-containing NMDARs by METTL14 overexpression (Figure 7, A–C). This interpretation was further supported by recordings of evoked EPSCs and PPR (Figure 7, D–G), which showed that bath application of TCN 201 reversed METTL14 overexpression–enhanced glutamate release via GluN2A-containing NMDARs. These results indicate that GluN2A is involved in METTL14-induced nociception and presynaptic NMDAR activation at the spinal cord level.

### METTL14-mediated m6A modification targets the Grin2a mRNA 3′-UTR.

**Figure 8:**
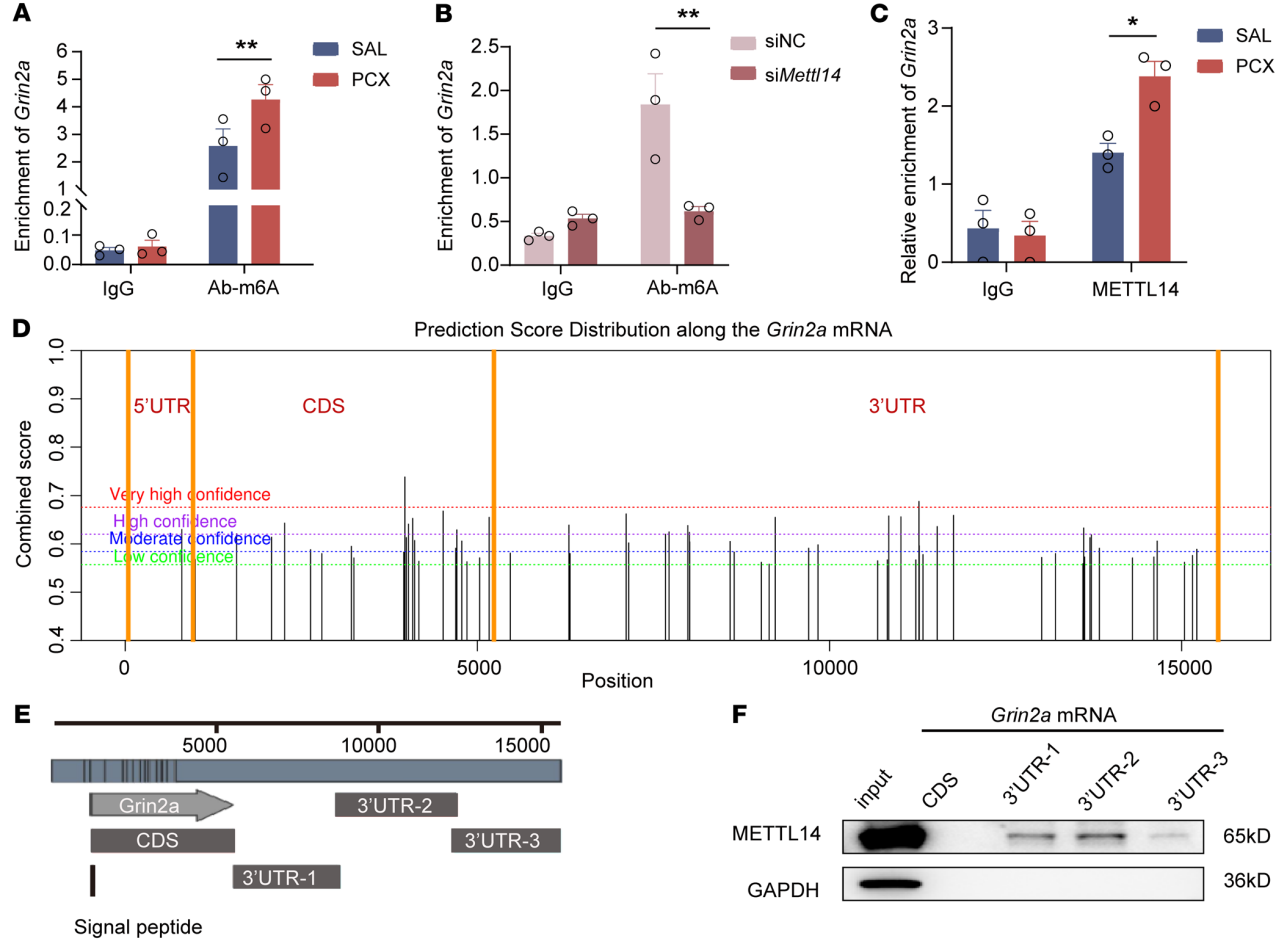
*Grin2a* is a downstream target of METTL14-mediated m6A modification after paclitaxel treatment. (**A**) RIP-qPCR results for enriched *Grin2a* mRNA in vivo using an m6A antibody compared with IgG control (*n* = 3 rats per group, 2-way ANOVA followed by Šidák’s post hoc test). (**B**) RIP-qPCR results for enriched *Grin2a* mRNA in rats after si*Mettl14* or scrambled siRNA intrathecal injection using an m6A antibody compared with IgG control (*n* = 3 rats per group, 2-way ANOVA followed by Šidák’s post hoc test). (**C**) RIP-qPCR results for enriched *Grin2a* mRNA in saline- or PCX-pretreated rats using a METTL14 antibody compared with IgG control (*n* = 3 rats per group, 2-way ANOVA followed by Šidák’s post hoc test). (**D**) SRAMP analysis was used for the prediction of m6A methylation sites in *Grin2a* mRNA. (**E**) Schematic illustration of biotin-labeled probes of *Grin2a* mRNA CDS (1224–5618 bp), 3′-UTR-1 (5618–8816 bp), 3′-UTR-2 (8792–12417 bp), and 3′-UTR-3 (12398–15635 bp). (**F**) Western blotting results for METTL14 protein pulled down by different probes described in **E**. The housekeeping gene GAPDH was used as an internal reference gene. Data are shown as mean ± SEM. *P < 0.05, **P < 0.01.

We attempted to determine how METTL14 controls *Grin2a* expression in the DRG. Considering the critical role of METTL14 in m6A methylation, we investigated whether METTL14 regulates *Grin2a* expression via m6A modification. To this end, we conducted RNA-binding protein immunoprecipitation using an m6A antibody and identified the coprecipitated RNA, followed by real-time PCR analysis. The results showed that the m6A antibody immunoprecipitation sample was highly enriched with *Grin2a* mRNAs in the DRG of paclitaxel-induced CINP rats compared with that in the controls (Figure 8A). Conversely, the enrichments of *Grin1* and *Grin2b* mRNAs in m6A precipitates were less and did not change after paclitaxel treatment (Supplemental Figure 6, A and B). To determine the role of METTL14 in paclitaxel-induced *Grin2a*-m6A enrichment, we assessed the effect of METTL14 knockdown using *Mettl14* siRNA and found that reducing METTL14 expression attenuated m6A modification–enriched *Grin2a* mRNA expression in the DRG (Figure 8B). In addition, immunoprecipitation using a METTL14 antibody showed that paclitaxel treatment consistently increased the binding of *Grin2a* mRNA fragments to METTL14 in the DRG (Figure 8C).

We used the SRAMP prediction server, a computational predictor of mammalian m6A sites (28), to determine potential m6A modifications of *Grin2a* mRNA. From the results, possible m6A modification sites of *Grin2a* mRNAs were mainly located in the coding sequence (CDS) and 3′-UTR regions (Figure 8D). However, few m6A modifications were predicted for *Grin1* and *Grin2b* mRNA (Supplemental Figure 6, C and D). We used an RNA pulldown assay to validate the predicted results for *Grin2a* mRNA in DRG tissues. Biotin-labeled probes of *Grin2a* mRNA CDS (1224–5618 bp), *Grin2a* mRNA 3′-UTR-1 (5618–8816 bp), *Grin2a* mRNA 3′-UTR-2 (8792–12417 bp), and *Grin2a* mRNA 3′-UTR-3 (12398–15635 bp) were synthesized (Figure 8E). The results showed that METTL14 was bound to *Grin2a* mRNA 3′-UTR-1 to -3 in the DRG, whereas the CDS probe enriched no METTL14 protein compared with that in the controls (Figure 8F). These results suggest that *Grin2a* is a downstream target of METTL14-mediated m6A modification in the DRG.

### IGF2BP2 contributes to Grin2a mRNA stability in an m6A-dependent manner.

**Figure 9:**
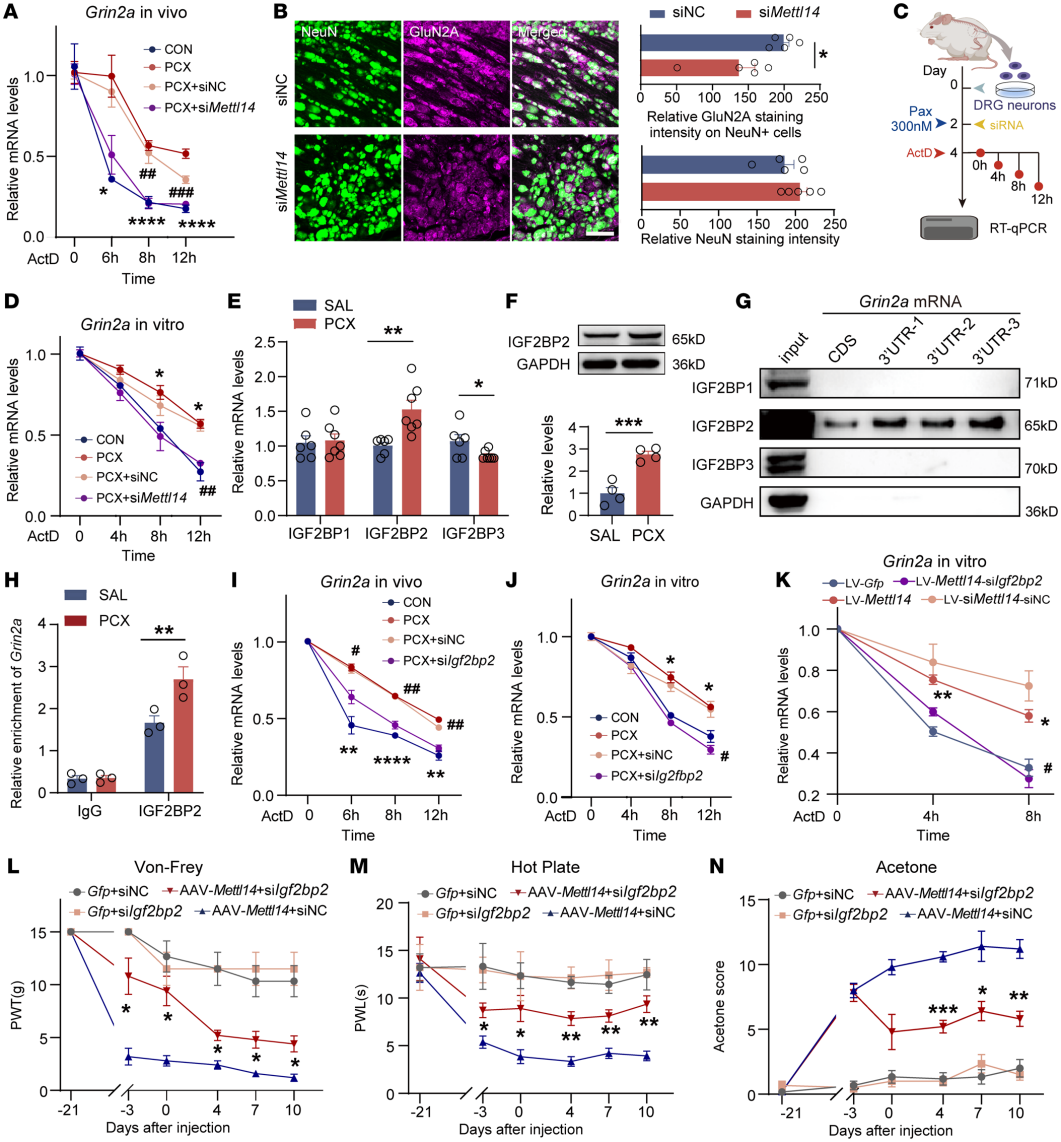
IGF2BP2 mediates the stability of *Grin2a* mRNA in an m6A-dependent manner and regulates pain hypersensitivity. (**A**) RNA stability curves in vivo (at least 4 rats per group; *PCX vs. CON, ^#^PCX+si*Mettl14* vs. PCX+siNC). (**B**) Double-labeled immunofluorescent images and quantification showing the colocalization and alteration of GluN2A in neurons. Bar plots show the relative GluN2A intensity in neurons and NeuN intensity (*n* = 5 rats per group). Scale bar: 50 μm. (**C**) Illustration of RNA stability assay in rat primary cultured DRG neurons. (**D**) RNA stability in cultured DRG neurons. (**E**) IGF2BP mRNA expression in DRGs in rats at day 10 (*n* = 6 rats per group, Student’s *t* test). (**F**) IGF2BP2 protein expression in DRGs at day 10 (*n* = 4, Student’s *t* test). (**G**) Western blotting results of IGF2BPs pulled down by different probes. (**H**) RIP-qPCR results for enriched *Grin2a* mRNA using an IGF2BP2 antibody compared with IgG control in saline- or PCX-pretreated rats (2-way ANOVA followed by Šidák’s post hoc test). (**I**) RNA stability curves in vivo (5 rats per group; *PCX vs. CON, ^#^PCX+si*Mettl14* vs. PCX+siNC). (**J**) RNA stability in cultured DRG neurons. (**K**) RNA stability in ActD-incubated PC12 cells transfected with lectin-*Mettl14* or lectin-*Gfp* combined with si*Igf2bp2* or scrambled siRNA (*lectin-*Mettl14* vs. the control, ^#^lectin-*Mettl14*+si*Igf2bp2* vs. lectin-*Mettl14*+siNC). (**L**–**N**) Mechanical allodynia (**L**), thermal hyperalgesia (**M**), and cold allodynia (**N**) of naive rats after AAV-*Mettl14* or AAV-*Gfp* combined with si*Igf2bp2* or scrambled siRNA intrathecal injection (*AAV-*Mettl14*+si*Igf2bp2* vs. AAV-*Mettl14*+siNC). Two-way ANOVA followed by Tukey’s post hoc test was used for RNA stability, whole-cell patch-clamp recording, and behavior data analysis. Data are shown as the mean ± SEM. *P < 0.05, **P < 0.01, ***P < 0.001, ****P < 0.0001; ^#^*P* < 0.05, ^##^*P* < 0.01, ^###^*P* < 0.001.

The regulatory role of the 3′-UTR in mRNA processing has been extensively studied (29). Importantly, the 3′-UTRs of mRNAs are essential to regulate RNA stability (30, 31). To determine whether paclitaxel treatment altered *Grin2a* mRNA stability, we conducted RNA half-life experiments using actinomycin D (ActD) and found that paclitaxel treatment enhanced the mRNA stability of *Grin2a* in PC12 cells (Supplemental Figure 7A). METTL14 knockdown reversed the enhanced stability induced by paclitaxel treatment and promoted *Grin2a* mRNA degradation (Supplemental Figure 7A). Next, we intrathecally injected ActD to inhibit transcription in vivo and evaluated the remaining *Grin2a* mRNA levels in DRG. Paclitaxel consistently increased the stability of *Grin2a* mRNA in the DRG, which was diminished by si*Mettl14* treatment (Figure 9A). Double immunofluorescence staining further indicated that *Mettl14* siRNA decreased GluN2A content in DRG neurons (Figure 9B). Importantly, the same tendency was observed in rat primary cultured DRG neurons (Figure 9, C and D). These results indicate a critical role of METTL14 in the maintenance of *Grin2a* mRNA stability in DRG neurons under CINP conditions.

Members of the IGF2BP family, including IGF2BP1, 2, and 3, are unique m6A readers capable of enhancing mRNA stability by recognizing m6A modification sites (12, 32, 33). Here, we found that paclitaxel treatment upregulated IGF2BP2, but not IGF2BP1 and IGF2BP3, in the DRG (Figure 9E). High IGF2BP2 protein expression in the DRG after paclitaxel treatment was also confirmed (Figure 9F). An RNA pulldown assay showed that IGF2BP2, but not other members of the IGF2BP family, bound explicitly to the *Grin2a* mRNA CDS and 3′-UTR regions and that the 3′-UTR interacted with more IGF2BP2 in the DRG (Figure 9G). RNA immunoprecipitation (RIP) assays in vivo revealed that the interaction between IGF2BP2 and *Grin2a* mRNAs in the DRG was enhanced by paclitaxel treatment (Figure 9H).

We then determined the role of IGF2BP2 in *Grin2a* mRNA stability maintenance. IGF2BP2 siRNA increased the degradation of *Grin2a* mRNAs in PC12 cells (Supplemental Figure 7, B and C). IGF2BP2 knockdown in vivo reversed the paclitaxel-induced high expression of *Grin2a*, and double immunolabeling further indicated that IGF2BP2 siRNA decreased the *Grin2a* content in DRG neurons (Supplemental Figure 7, D and E). Importantly, RNA stability assays in vivo showed that IGF2BP2 siRNA decreased the stability of *Grin2a* mRNA in the DRG (Figure 9I), and the same tendency was observed in rat primary cultured DRG neurons (Figure 9J). Moreover, IGF2BP2 siRNA also notably disrupted the stability of *Grin2a* mRNAs directly induced by METTL14 overexpression (Figure 9K). These data demonstrated the important role of IGF2BP2, an m6A reader, in the maintenance of *Grin2a* mRNA stability in DRG neurons.

Given the important role of GluN2A-containing NMDARs in CINP, we investigated whether IGF2BP2 plays a role in METTL14 overexpression–induced hypersensitivity and CINP. Behavioral data showed that IGF2BP2 knockdown via IGF2BP2 siRNA markedly ameliorated tactile hyperalgesia, thermal allodynia, and cold pain induced by METTL14 overexpression (Figure 9, L–N) and paclitaxel treatment (Supplemental Figure 7, F–H) in naive rats. Taken together, these findings suggest that IGF2BP2 contributes to *Grin2a* mRNA stability in the DRG neurons and pain hypersensitivity caused by METTL14 overexpression and paclitaxel treatment.

### D-box–binding PAR BZIP transcription factor elevates METTL14 in CINP.

**Figure 10:**
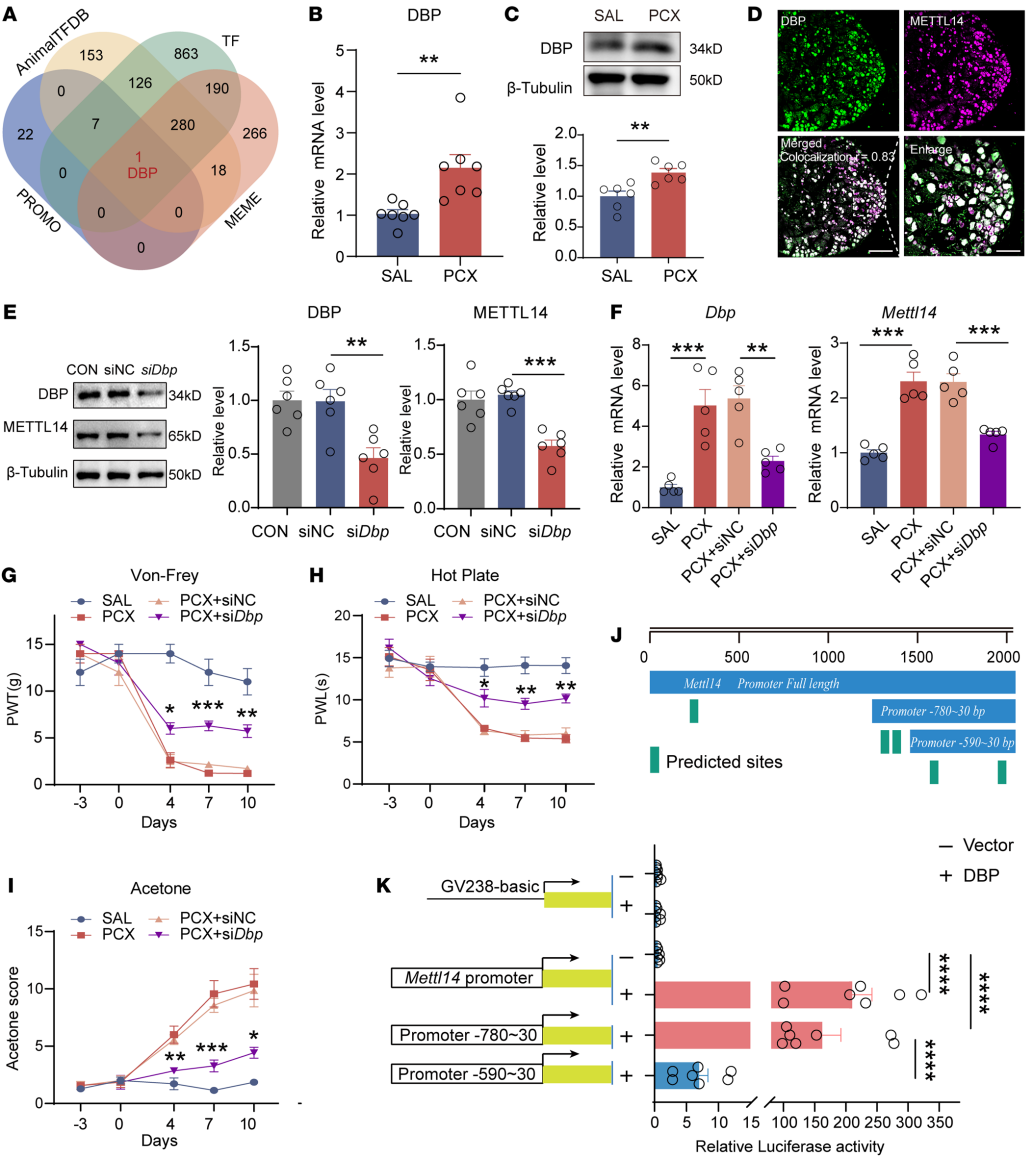
The transcription factor DBP mediates METTL14 upregulation caused by paclitaxel treatment. (**A**) Venn diagram shows the predictive transcription factors (TFs) by taking intersections with the TFs of the rat species and predictive results from the PROMO, AnimalTFDB, and MEME (FIMO function) databases. (**B** and **C**) *Dbp* mRNA (**B**) and protein (**C**) expression in the bilateral L4–L6 DRGs in saline- or PCX-pretreated rats (at least 6 rats per group, Student’s *t* test). (**D**) Colocalization analysis of DBP and METTL14 based on double-labeled immunofluorescent images. Scale bars: 100 μm, left; 50 μm, enlarged image. (**E**) DBP and METTL14 protein expression in PC12 cells transfected with si*Dbp* compared with the control (*n* = 6 per group, 1-way ANOVA followed by Tukey’s post hoc test). (**F**) *Dbp* and *Grin2a* mRNA expression in the rat bilateral L4–L6 DRGs in saline- or PCX-treated rats with siRNA-*Mettl14* or scrambled siRNA injection (*n* = 5 rats per group, 1-way ANOVA followed by Tukey’s post hoc test). (**G**–**I**) Mechanical allodynia, thermal hyperalgesia, and cold allodynia of saline- or PCX-treated rats after siRNA-Dbp or scrambled siRNA treatment (at least 7 rats per group, 2-way ANOVA followed by Tukey’s post hoc test). (**J**) Top: Schematic representation of the full-length *Mettl14* promoter, truncation promoters (–780 bp to ~30 bp, –590 bp to ~30 bp), and predicted binding sites from the JASPAR database. Bottom: Vectors containing full-length *Mettl14* promoter and *Mettl14* truncation promoters (–780 bp to ~30 bp) show higher luciferase activity (at least *n* = 7 per group, 1-way ANOVA followed by Tukey’s post hoc test). Data are shown as the mean ± SEM. *P < 0.05, **P < 0.01, ***P < 0.001, ****P < 0.0001.

Finally, we determined how paclitaxel treatment increased METTL14 expression in the DRG. We initially used 3 databases, PROMO, Animal Transcription Factor Database (AnimalTFDB), and MEME, to identify the potential transcription factors (TFs) for the *Mettl14* promoter. By intersecting with the TFs of rat species, the D-box–binding PAR BZIP transcription factor (DBP) was the only predicted TF binding to the promoter of *Mettl14* (Figure 10A). qPCR and immunoblotting analyses showed that paclitaxel treatment increased DBP expression in the DRG (Figure 10, B and C). Furthermore, the colocalization of DBP with METTL14 in the DRG suggested the potential involvement of DBP in the regulation of METTL14 expression (Figure 10D). We screened and selected the most effective siRNA sequence targeting *Dbp* (Supplemental Figure 8A) and validated the role of DBP in the regulation of METTL14 expression. Results showed that DBP knockdown markedly reduced METTL14 expression both in vivo and in vitro (Figure 10, E and F). Double immunofluorescence staining showed that si*Dbp* decreased METTL14 content in DRG neurons (marked by the NeuN marker) (Supplemental Figure 8B). Considering the functional role of METTL14 in CINP, we investigated whether DBP knockdown reversed the paclitaxel-induced nociceptive behaviors. As expected, the si*Dbp* intrathecal injection attenuated paclitaxel-induced mechanical hypersensitivity (Figure 10G), heat hyperalgesia (Figure 10H), and cold allodynia (Figure 10I).

Next, we sought to determine the binding sites of DBP to the *Mettl14* promoter. We analyzed the promoter sequence of *Mettl14* using the JASPAR database and found more than 5 putative binding sites (Figure 10J and Supplemental Figure 8C). Dual-luciferase reporter assays were performed to confirm this interaction in the transfected HEK293T cells. The results showed that the full-length *Mettl14* promoter and truncated promoter (–780 bp to ~30 bp) vector, but not the control vector or truncated promoter (–590 bp to ~30 bp), dramatically enhanced the luciferase activity of the *Mettl14* promoter driven by the full-length DBP vector (Figure 10K). These results show that DBP regulates the transcription of *Mettl14* by binding to its promoter (mainly located at –780 bp to –590 bp), thereby contributing to the METTL14 expression and pain sensitization in CINP.

## Discussion

This study provides the first evidence to our knowledge that METTL14, an m6A methyltransferase, plays a key role in CINP via m6A modification and upregulation of GluN2A in the DRG. METTL14 is a critical RNA-binding scaffold in the m6A methyltransferase complex that acts at the post-transcriptional level (34). Sharing 43% identity with METTL3, METTL14 enhances the catalytic activity of METTL3 as a homolog (35). Previous studies have demonstrated the importance of METTL3 in nociception. Zhang et al. found that METTL3 is downregulated in the spinal cord of spared nerve injury (SNI) models, which accelerates microRNA-150primary afferent terminal maturation and contributes to nociception (36). He et al. showed that METTL13-mediated m6A modification could also target long noncoding RNA D26496 and induce its degradation in sciatic nerve segments (37). However, the role of METTL14 in CINP remains unclear.

Recent evidence has demonstrated a crucial role for METTL14 in diverse physiological and pathological processes, including sciatic nerve lesions, oligodendrocyte maturation, and CNS myelination (38). In the present study, we demonstrated that paclitaxel treatment increased METTL14 expression, contributing to m6A modification in the DRG. High METTL14 expression has also been observed in human DRG treated with chemotherapeutic agents. Functionally, silencing METTL14 expression dramatically reversed tactile, thermal, and cold hypersensitivity induced by paclitaxel treatment, whereas METTL14 overexpression induced mechanical, thermal, and cold hypersensitivity, suggesting that METTL14 plays an essential role in CINP.

Increased presynaptic NMDAR activity at the spinal dorsal horn level is an important feature of CINP (21, 23). Paclitaxel-mediated enhancement of presynaptic NMDAR activity preferentially amplifies primary afferent inputs to spinal excitatory dorsal horn neurons. The ablation of NMDARs in DRG neurons completely blocks the development of paclitaxel-induced neuropathic pain (39). In this study, we consistently found a notable increase in the mEPSC frequency and amplitude of EPSCs of spinal dorsal horn neurons evoked from the dorsal root in paclitaxel-treated rats, which could be normalized by AP5, an NMDAR antagonist, suggesting a role of NMDAR-mediated glutamatergic input from primary afferents in CINP. PPR recording further supported the presynaptic action in CINP. As METTL14 is predominantly expressed in DRG neurons but not in satellite glial cells or macrophages, we determined whether METTL14 was involved in the associated neuronal activity. Surprisingly, we found that silencing METTL14 reversed these high preNMDAR activities in paclitaxel-treated rats, whereas increasing METTL14 expression in naive rats recapitulated the effects of paclitaxel at the electrophysiological level. These findings reveal an important role of METTL14 in presynaptic NMDAR activation in CINP.

Paclitaxel-induced glutamatergic input to spinal dorsal horn neurons is predominantly mediated by presynaptic GluN2A-containing NMDARs (21). Additionally, selective inhibition of GluN2A in the DRG reduces hypersensitivity caused by inflammation and SNI (40), and GluN2A conditional knockout in the trigeminal ganglion prevents mechanical allodynia induced by inferior alveolar nerve transection (41). We showed that paclitaxel treatment increased GluN2A expression in the DRG, which was positively correlated with increased METTL14 levels after paclitaxel treatment. We used gain- and loss-of-function strategies to determine the role of METTL14 in the control of GluN2A expression. METTL14 silencing reversed the paclitaxel-induced increase in GluN2A expression in the DRG. Conversely, mimicking the increased METTL14 expression in naive rats markedly increased GluN2A expression. In addition, TCN 201, a selective GluN2A-containing NMDAR antagonist, attenuated presynaptic NMDAR hyperactivity and mechanical, thermal, and cold hypersensitivity caused by METTL14 overexpression in rats. These results strongly suggest that METTL14 mediates paclitaxel-induced presynaptic NMDAR hyperactivity and pain hypersensitivity via GluN2A.

Epigenetic modifications of NMDARs have been highlighted in the control of neuronal function. Aberrant GRIN2A hypermethylation has been found in the hippocampus and prefrontal cortex in major depressive disorder (42). Palmitoylation of NMDAR subunits differentially modulates receptor trafficking and may be critical for synaptic plasticity (43). Considering the role of METTL14 in m6A modification and the colabeling results indicating that METTL14 was mainly located in the nucleus, we determined whether METTL14 regulates GluN2A via m6A modification. Paclitaxel treatment caused marked m6A modification of *Grin2a* mRNA in the DRG. SRAMP analysis also indicated that more potential m6A-modified sites were present in the *Grin2a* mRNA than in *Grin1* or *Grin2b*. Using RIP and RNA pull-down assays, we found that the METTL14-*Grin2a* interaction was primarily in the *Grin2a* mRNA 3′-UTR.

RNA stability is critical for nociception. *N*^4^-acetylcytidine modification of *Syt9* mRNA in the DRG enhances its stability and triggers nerve injury–induced nociceptive hypersensitivities (44). LINC01119 contributes to SNI-induced neuropathic pain by stabilizing the BDNF transcript at the spinal cord level (45). Additionally, m6A marks influence modified transcripts in diverse ways by recruiting different m6A readers and regulating the fate of RNA (46). m6A hypomethylation of DNMT3B enhances its expression in the intervertebral discs and promotes low back pain (47). The demethylase FTO in the spinal dorsal horn promotes nociception by regulating m6A methylation of CXCR3 and increasing mRNA stability (48). Considering the role of the 3′-UTR in mRNA stability, we determined whether METTL14-mediated m6A modification affects *Grin2a* mRNA stability. We found that paclitaxel treatment enhanced the mRNA stability of *Grin2a* in the DRG, which was reversed by METTL14 knockdown. To further define the mechanisms involved, we identified proteins responsible for stability maintenance. Paclitaxel treatment caused a large increase in IGF2BP2 expression in the DRG. Furthermore, RIP and RNA pulldown assays showed the interaction between IGF2BP2 and the 3′-UTR of *Grin2a*. Using a loss-of-function approach, we found that IGF2BP2 enhanced the stability of *Grin2a*. In addition, IGF2BP2 knockdown markedly attenuated the pain hypersensitivity induced by METTL14 overexpression and paclitaxel treatment. As a crucial member of the IGF2BP family, IGF2BP2 regulates mRNA stability by targeting GGC (m6A) sequences (49). Our study identified IGF2BP2 as a critical m6A reader protein that directly binds to a specific site on *Grin2a*, thereby regulating the half-life of *Grin2a* mRNA under CINP conditions.

TFs are specialized proteins that bind to nucleosomal DNA and regulate transcription (50). Several TFs may be involved in the regulation of m6A levels. For instance, the zinc finger protein 217 (Zfp217) is crucial for adipogenic differentiation by activating the transcription of the m6A demethylase FTO (51). HIF-1α, a major TF under hypoxic conditions, regulates YTHDF1 transcription by binding to its promoter region, thus driving malignancy (52). In this study, we predicted and confirmed potential regulatory TFs that contribute to the upregulation of METTL14 in CINP. We screened and validated DBP as an upstream TF of *Mettl14*. The *Mettl14* gene is transcriptionally activated through DBP upregulation in paclitaxel-treated DRGs. DBP silencing markedly reduced *Mettl14* mRNA and protein expression. A dual-luciferase reporter assay further confirmed the DBP–*Mettl14* gene interaction, which is mainly located at –780 bp to –590 bp in the promoter of *Mettl14*. Thus, DBP is an important TF responsible for METTL14 upregulation in CINP.

Though we have extensively studied and disclosed the relation between METTL14-mediated m6A and GluN2A in neuropathic pain, several limitations should be noted. Firstly, the functional role of METTL14 in DRG neurons has not been explicitly defined and needs to be validated in mice with DRG neuron–specific conditional KO or by use of CRISPR/Cas9 editing. And the sex-dependent effect of GluN2A inhibition using TCN 201, and IGF2BP2 knockdown using siRNA, should be further assessed. Secondly, the m6A-modified sites on *Grin2a* mRNA should be studied further. Additionally, although we demonstrated the essential role of METTL14-mediated m6A modification in targeting presynaptic GluN2A in CINP, other potential epigenetic mechanisms, such as DNA methylation and *N*^4^-acetylcytidine modification of GluN2A, cannot be ruled out and require further investigation.

In conclusion, our findings reveal that METTL14-mediated m6A modification plays a major role in GluN2A expression in an IGF2BP2-dependent manner, enhancing its stability in DRG neurons and contributing to presynaptic NMDAR-mediated glutamatergic input to spinal dorsal horn neurons in CINP. Our findings provide new evidence regarding the importance of RNA m6A modifications in the control of synaptic plasticity and neuropathic pain, and suggest new targets to treat CINP.

## Methods

### Sex as a biological variable

Our study examined male and female patients and animals. Similar findings were reported for both sexes.

### Patient DRG tissues

Human DRG tissues treated with or without chemotherapeutic agents were retrieved from patients who underwent surgery at the Sun Yat-sen University Cancer Center for tumor metastasis and invasion of the vertebral body. Fresh DRG tissues were washed with saline after resection, embedded in OCT compound, and kept at –80°C for later use. The clinical features of the patients are presented in Supplemental Table 1.

### Animals

Male and female Sprague-Dawley rats (40–80 g and 220–250 g) were purchased from Guangdong Medical Laboratory Center (Guangzhou, China). The rats were housed in a temperature- and humidity-controlled environment (12-hour light/12-hour dark cycle, with water available ad libitum).

### CINP model

A rat model of CINP was established as previously described (21). Briefly, 6 mg/mL stock pharmaceutical-grade paclitaxel (Taxol, C47H51NO14, Bristol Myers Squibb) was diluted with sterile 0.9% saline to 1 mg/mL and intraperitoneally injected into rats at a dose of 2 mg/kg every 4 days (days 1, 3, 5, and 7; a total cumulative dose of 8 mg/kg). Saline was injected as a vehicle control.

### Behavior tests

#### Von Frey assay.

Von Frey filaments (Stoelting) were used to measure the mechanical paw withdrawal threshold as previously described (53, 54). Briefly, the rats were placed in 20 × 20 cm Plexiglas boxes with a metallic mesh floor 20 cm above the bench. Different calibrated monofilaments, ranging from 0.4 to 15 g, were applied for up to 3 seconds in an ascending manner to the mid-plantar area of the right hind paw with sufficient force to bend the fiber. Each rat was stimulated with each filament 5 times, with each interval lasting at least 3 minutes. Paw withdrawal responses were recorded. Sudden claw retraction, shaking, and licking were considered positive reactions. Paw withdrawal threshold was defined as the minimum force (*g*) required to induce a positive response at least 3 times in 5 tests (55).

#### Hot plate test.

A hot plate test was conducted to measure thermal hypersensitivity using an Xr1700 intelligent instrument (55°C) (Shanghai Softmaze Information Technology Co.). Briefly, the rats were placed on a hot plate, and a timer controlled by a foot pedal began timing the response latency. The time from when the rat licked its hind paw to when it jumped off the hot plate was recorded as nociceptive latency. A cutoff time of 60 seconds was used to avoid scalding.

#### Acetone test.

Cold allodynia was assessed using the acetone test. Rats were acclimated to 20 × 20 cm Plexiglas boxes for at least 30 minutes, and 1 mL of acetone was then applied to the soles of the rats. The rats were observed for 30 seconds after acetone stimulation. Cold allodynia–like behavior in the hind paw was evaluated by scoring of the responses after 5 stimuli according to the following 4-point scale: 0, no response; 1, shift; 2, withdrawal; 3, flinching or licking; and 4, vocalization. Each hind paw was tested every 5 minutes, and the total reaction score of the rats after 5 stimulations was used as the acetone score for cold allodynia.

### Rat DRG tissue collection

Rats were anesthetized with sodium pentobarbital (100 mg/kg) and then perfused with 150 mL cold 0.9% saline through the intracardiac route. After perfusion, the DRGs (L4–L6) were collected.

### Dot blot assay

Total RNA from the DRGs was harvested using TRIzol reagent (Invitrogen). A total of 400 ng RNA per group (1 μL) was transferred onto a nitrocellulose membrane (Amersham, GE Healthcare), and the membranes were then UV-cross-linked, blocked, and incubated with a specific m6A antibody (1:1,000; Synaptic Systems). The other membranes were stained with 0.02% methylene blue as a loading control. Dot blot pixel density values were determined by ImageJ software (NIH). Relative density was corrected and normalized to the control loading.

### PC12 culture and transfection

PC12 cells (ATCC) were routinely cultured in DMEM (Gibco) supplemented with 5% horse serum and 5% FBS (Gibco) and incubated in a 5% CO_2_, 37°C incubator. siRNAs directed against METTL14, IGF2BP2, and DBP and negative control RNAs (siNC) were synthesized by RiboBio. Transient transfection was performed using a RiboFECT CP Transfection Kit (RiboBio) according to the standard protocol. The siRNAs used in this study are listed in Supplemental Table 2.

### Primary DRG neuron culture

Sprague-Dawley rats (40–80 g) were anesthetized with sodium pentobarbital (100 mg/kg), and the DRGs were quickly removed from the dorsal root and collected in a cold neurobasal medium (Thermo Fisher Scientific) containing 10% FBS. The DRGs were then cut into pieces and treated with 3 mg/mL collagenase I (20 minutes), followed by 0.25% trypsin-EDTA (10 minutes) in a 95% O_2_, 5% CO_2_, and 37°C incubator. During digestion, the tissue suspension was gently blown at 10-minute intervals using a 1 mL pipette tip. After trypsinization termination and centrifugation at 143*g* for 5 minutes, the supernatant was discarded, and the dissociated neurons were resuspended in a mixed neurobasal medium containing 10 % FBS, B-27 supplement (Thermo Fisher Scientific), GlutaMAX Supplement (Invitrogen), 100 U/mL of penicillin, and 100 mg/mL of streptomycin. The neurons were seeded in a 12-well plate coated with poly-l-lysine (PLL) (MilliporeSigma) and cultured for 48 hours.

### siRNA and recombinant adeno-associated virus administration

siRNAs targeting METTL14, IGF2BP2, and DBP and siRNA negative control were obtained from RiboBio. Intrathecal injection of siRNA (4 nmol, 20 μL) was performed once every 3 days in control or CINP rats. Recombinant AAV9 encoding METTL14 (AAV9-*Mettl14*) and recombinant AAV9 encoding GFP (AAV9-*Gfp*) were obtained from GeneChem Co. Ltd. AAV9 was injected intrathecally 21 days before the first intraperitoneal injection of paclitaxel.

### Intrathecal injection

Under isoflurane anesthesia, rats were placed horizontally on the platform. A 30-G needle attached to a 25 μL microsyringe was inserted between the L5 and L6 vertebrae. After the needle was inserted into the subarachnoid space, a wag or slight flick of the tail indicated that the needle was positioned correctly.

### Real-time qPCR

Total RNA from cells or tissues was extracted using TRIzol reagent (Invitrogen) and reverse-transcribed into complementary DNA. Quantitative PCR (qPCR) analysis was performed using a Real-Time Detection System with Color SYBR Green qPCR Mix (EZBioscience). GAPDH was used as the internal control for normalization. The 2^−ΔΔCt^ method was used to calculate mRNA levels. All primers used are listed in Supplemental Table 3.

### Western blotting

Cultured cells or bilateral L4–L6 DRGs were lysed in ice-cold lysis buffer (10 mM Tris, 5 mM EGTA, 2 mM MgCl_2_, 1 mM DTT, 1 mM PMSF, and 40 μM leupeptin). After centrifugation (4°C, 15 minutes, 12,000*g*), the supernatants were measured and then heated at 100°C for 5 minutes. Total protein samples (25 μg per lane) were loaded and separated by 10% SDS-PAGE and transferred to PVDF membranes. The membranes were blocked with 5% nonfat milk for 1 hour and then incubated overnight with primary antibodies against METTL14 (1:2,000; A8530, ABclonal), GluN2A (1:1,000; 4205S, Cell Signaling), GluN2B (1:1,000; 21920-1-AP, Proteintech), GluN1 (1:1,000; A7677, ABclonal), FTO (1:1,000; 45980S, Cell Signaling), METTL3 (1:1,000; 15073-1-AP, Proteintech), METTL16 (1:2,000; 17676S Cell Signaling), WTAP (1:1,000; 60188-1-Ig, Proteintech), ALKBH5 (1:1,000; 16837-1-AP, Proteintech), IGF2BP1 (1:1,000; A1517, ABclonal), IGF2BP2 (1:1,000; 11601-1-AP, Proteintech), IGF2BP3 (1:1,000; A6099, ABclonal), DBP (1:1,000; 12662-1-AP, Proteintech), GAPDH (1:5,000; 60004-1-Ig, Proteintech), and β-tubulin (1:5,000; AC008, ABclonal). The following day, the membranes were washed and incubated with horseradish peroxidase–labeled goat anti-rabbit IgG (1:10,000; SA00001-2, Proteintech) or horseradish peroxidase–labeled goat anti-mouse IgG (1:10,000; 7076S, Cell Signaling). Immune complexes were detected using chemiluminescence, and ImageJ software was used to quantify the bands. The bands for each protein were normalized to the respective GAPDH or β-tubulin loading controls.

### RNA stability assay

PC12 cells were exposed to paclitaxel (15 ng/mL,48 hours) or saline control and transfected with si*Mettl14*, si*Igf2bp2*, lectin-*Mettl14*, and other controls. The cells were then treated with 5 μg/mL actinomycin D (ActD) (HY-17559, MedChemExpress) for 2, 4, 6, and 8 hours, and the cells were collected. Subsequently, mRNA levels were quantified by real-time qPCR.

Primary DRG neurons were treated with 300 nM paclitaxel or saline in a mixed neurobasal medium for 48 hours. si*Mettl14*, si*Igf2bp2*, and siRNA controls were transfected into cultured neurons using Lipofectamine RNAiMAX (Invitrogen). Neurons were then collected at 0-, 4-, 8-, and 12-hour time points after 1 mg/mL ActD addition for qPCR.

RNA stability assessment in vivo was conducted as previously described (56). Briefly, 37.5 μg/100 g ActD was intrathecally injected to inhibit transcription, and the DRGs from different groups were collected at 0-, 6-, 8-, and 12-hour time points after ActD treatment for quantification.

### Immunofluorescence

For rat tissues, rats were anesthetized and intracardially perfused with saline, followed by ice-cold 4% paraformaldehyde (PFA). The L4–L6 DRGs or spinal dorsal horns were quickly removed and postfixed with 4% PFA, equilibrated in 30% sucrose, and cut into 15-μm (for DRG) or 20-μm (for spinal dorsal horn) frozen sections. For human DRG tissues, pre-embedded DRGs were cut into 30-μm frozen sections. All sections were blocked with PBS containing 5% goat serum at 26°C for 1 hour and then incubated overnight at 4°C with the following primary antibodies: rabbit anti-METTL14 (1:200; A8530, ABclonal), mouse anti-METTL14 (1:200; CL4252, Abcam), mouse anti-NeuN (1:100; MAB377, Millipore), goat anti-GFAP (1:300; ab53554, Abcam), mouse anti-Iba1 (1:300; ab5076, Abcam), mouse anti–glutamine synthetase (1:200; MA5-27749, Invitrogen), mouse anti-CGRP (1:200; ab81887, Abcam), mouse anti-NF200 (1:200; N0142, MilliporeSigma), mouse anti-IB4 (1:50; L2895, MilliporeSigma), rabbit anti-DBP (1:200; 12662-1-AP, Proteintech), and mouse anti-FLAG (1:1,000; 66008-4-Ig, Proteintech). After washing, the sections were incubated with the corresponding secondary antibodies conjugated to cyanine 3 (Cy3) (mouse, 1:400, 705-165-003, Jackson ImmunoResearch; rabbit, 1:500, HA1102, HUABIO), Alexa Fluor 488 (mouse or rabbit, 1:500; HA1121 and HA1125, HUABIO), or Alexa Fluor 647 (rabbit, 1:500; HA11253, HUABIO) at room temperature for 1 hour in the dark and coverslipped on the DAPI-containing sealing tablet (S2110, Solarbio) for observation. The stained section images were then captured by a Zeiss 980 confocal microscope or Nikon fluorescence microscope camera. To eliminate issues with red-green color blindness, we present red fluorescence in images as magenta. ImageJ software was used to determine the relative immunofluorescence intensity in a double-blind manner in selected DRG. The mean gray value depicting the level of immunofluorescence expression intensity for the target protein was measured. ROI Manager tool was used to filter the METTL14 and GluN2A expressed in neurons. For double-labeling immunofluorescence analysis, colocalization counts were measured in a double-blind manner, and colocalization of DBP and METTL14 signals was analyzed by the plug-in Coloc 2.

### RNA immunoprecipitation assay

An RIP Kit (IEMed-K303, IEMed) was used for the RNA immunoprecipitation (RIP) assay. Tissues of DRGs (0.1–0.2 g) were collected for each sample and then mechanically sheared in RIP lysis buffer containing a protease inhibitor cocktail and RNase inhibitor using a homogenizer. After centrifugation (4°C, 15 minutes, 12,000*g*), the supernatant was collected and split into 3 fractions: input, IP, and IgG. Antibodies against m6A (3 μg; Synaptic Systems), METTL14 (3 μg; ABclonal), normal rabbit IgG, and Protein A/G beads were added to the supernatant and incubated at room temperature for 1 hour. Beads were collected and washed 5 times with RIP washing buffer, and the RNA was eluted from the beads by addition of 20 μL RNase-free water. Purified RNA was subjected to qPCR to determine the presence of binding targets.

### RNA pull-down

RNA pull-down was performed using an RNA pull-down kit (IEMed-K302, IEMed), and biotinylated GluN2A probes and negative control probes were designed and synthesized by IEMed. The probes (2 μg) were denatured at 90°C for 2 minutes, which can form RNA secondary structures, and then incubated with streptavidin magnetic beads for 30 minutes at 25°C. DRG tissues (0.1–0.2 g) were homogenized and lysed using RIP buffer with a protease inhibitor cocktail. After centrifugation (4°C, 10 minutes, 12,000*g*), the supernatant was collected and incubated with streptavidin magnetic beads bound to biotinylated probes for 2 hours at 25°C with rotation. Then, pulled-down proteins were analyzed by Western blotting, following the denaturation of the bead-probe-protein complex in protein elution buffer at 95°C.

### Transcription factor prediction

To identify the potential upstream TF for *Mettl14*, we intersected 1,466 previously identified TFs in rats (57) with predictive results from 3 online databases — PROMO, Animal Transcription Factor Database (AnimalTFDB), and MEME (FIMO function) — based on the sequence of the *Mettl14* promoter.

### Luciferase reporter assay

The promoter sequence of *Mettl14* was obtained from GenBank (NM_001399249). The JASPAR online database was used to predict the DBP-binding sites on the *Mettl14* promoter. The full-length *Mettl14* promoter and truncated promoter (–780 bp to ~30 bp, –590 bp to ~30 bp) were synthesized separately by Shanghai Jikai Gene Chemical Technology. PmirGlo luciferase reporter vectors containing firefly luciferase (F-luc) and Renilla luciferase (R-luc) were purchased from Promega. The full-length *Dbp* transcript was inserted into a vector containing the F-luc coding sequence to construct a wild-type DBP reporter plasmid. Full-length *Mettl14* or truncated promoters were inserted to construct wild-type or mutated *Mettl14* promoter plasmids. HEK293T cells were seeded in 24-well plates and transfected with wild-type or mutated F-luc–*Mettl14* fusion reporter plasmid combined with the F-luc–*Dbp* fusion reporter plasmid via the lipo3000 reagent (Thermo Fisher Scientific). Twenty-four hours after transfection, the luciferase activity was measured using the Dual-Luciferase Reporter Assay System (E1910, Promega). R-luc activity was used as the control to normalize the transfection efficiency of the reporter plasmids.

### Whole-cell patch-clamp recording

Spinal cord slices were prepared as described previously (20). Briefly, rats were anesthetized, and the L4–L6 spinal cord tissues were collected. Artificial cerebrospinal fluid (ACSF), including 234 mM sucrose, 12.0 mM glucose, 1.2 mM NaH_2_PO_4_, 3.6 mM KCl, 1.2 mM MgCl_2_, 2.5 mM CaCl_2_, and 25.0 mM NaHCO_3_, was ice-cooled and oxygenated before use. The spinal cord tissues were placed in the ACSF immediately after resection. A vibratome (Leica Microsystems) was used to slice the spinal cord into 400-μm sections. Pre-oxygenated Krebs solution (containing 11.0 mM glucose, 117.0 mM NaCl, 3.6 mM KCl, 1.2 mM MgCl_2_, 25.0 mM NaHCO_3_, 2.5 mM CaCl_2_, and 1.2 mM NaH_2_PO_4_) was used to preincubate the slices at least 1 hour before recording at 34°C.

Each spinal cord slice was fixed in a glass chamber with Krebs solution continuously perfused at 34°C and 5.0 mL/min. Lamina II outer zone neurons (preferentially receiving nociceptive input from primary afferent nerves; ref. 22) were visualized and selected for substantial recording using an upright fixed-stage microscope with differential interference contrast/infrared illumination to determine the associated electrophysiological activity in the primary afferent nerves and spinal cord. Miniature EPSCs (mEPSCs) of lamina II neurons and evoked monosynaptic excitatory postsynaptic currents (eEPSCs) in response to 20 Hz electrical stimulation were identified and recorded as previously described (24). Additionally, to better measure the paired-pulse ratio (PPR), multiple eEPSCs were evoked independently and recorded at 50-millisecond intervals.

All drugs used in the recordings were freshly prepared and dissolved in ACSF. 2-Amino-5-phosphonopentanoic acid (AP5; 50 μM), a specific NMDAR antagonist, was purchased from HelloBio. TCN 201, a selective GluN2A-containing NMDAR antagonist, was purchased from MedChemExpress. At least 3 rats were used for each recording protocol.

### Statistics

All statistical analyses were performed using GraphPad Prism software (version 9.0). Data are presented as the mean ± SEM. The statistical significance of the differences was analyzed using a 2-tailed Student’s *t* test or 1- or 2-way ANOVA followed by multiple post hoc tests. The statistical details can be found in the legends for each figure. All experiments were independently performed in triplicate, and *P* less than 0.05 was regarded as a statistically significant difference. Although a power analysis was not performed, the sample size was based on previous reports and pilot studies conducted in this field.

### Study approval

This study conformed to the National Institutes of Health guidelines for the care and ethical treatment of animals, and all experimental procedures were approved by the Sun Yat-sen University Cancer Center Animal Care and Use Committee. Written informed consent was obtained from all participants for human DRG. This study was approved by the Ethics Committee of Sun Yat-sen University Cancer Center. All tests were conducted in a double-blind manner.

### Data availability

The data analyzed in this study are available upon reasonable request. Values for all data points in the plots are provided in the Supporting Data Values file.

## Author contributions

JX, HM, and JH conceived and designed the study. WL, XY, W Zhong, and GC did most of the experiments. WL, XY, and XG contributed to whole-cell patch-clamp recording. QY, YX, ZQ, YY, JZ, YW, XW, SW, and QZ assisted in carrying out the experiments. WL, XY, W Zhong, GC, and XG contributed to data analysis. WL and XY prepared and revised the figures and drafted the manuscript. JX, HM, W Zeng, and JH supervised the research. All authors read and approved the final manuscript.

## Supplementary Material

Supplemental data

Unedited blot and gel images

Supporting data values

## Version 1 02/06/2024

In-Press Preview

## Version 2 03/15/2024

Electronic publication
