# Supplementary material for: METTL14-mediated m6A epitranscriptomic modification contributes to chemotherapy-induced neuropathic pain by stabilizing GluN2A expression via IGF2BP2
Source: J Clin Invest. 2024 Feb 6;134(6):e174847. doi: 10.1172/JCI174847 (PMC10940092; doi:10.1172/JCI174847)
Supplement: Supplemental data [file jci-134-174847-s176.pdf]

Supplemental Figures

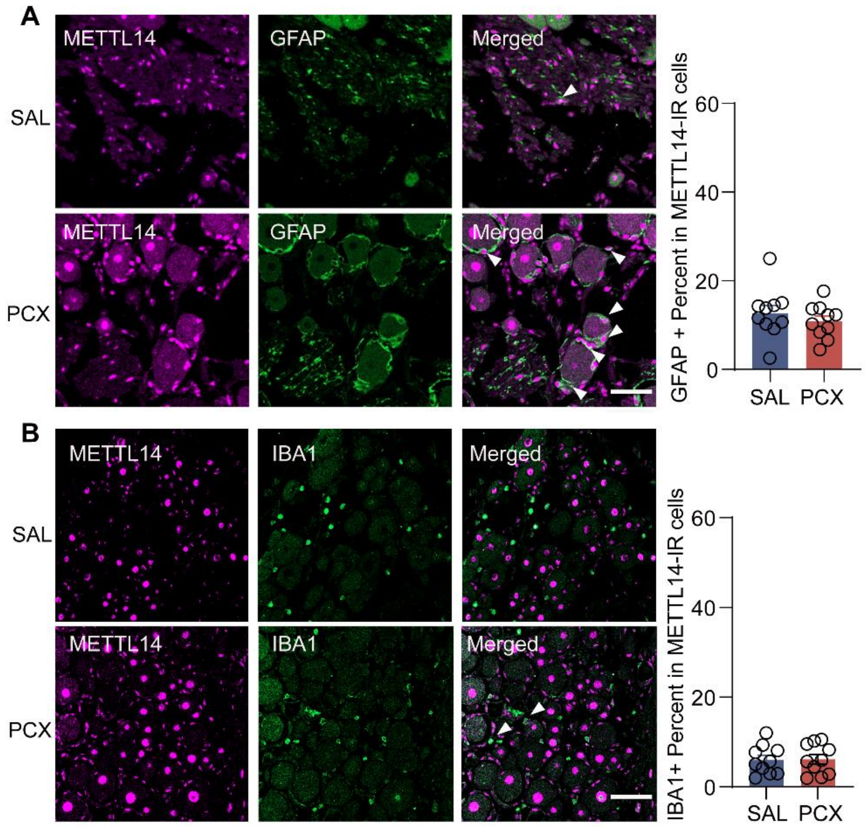

Supplemental Figure 1. Colocalization of METTL14 and GFAP, IBA1 in rat DRG. (A, B) Representative double-immunofluorescence staining and quantification showing colabeling of METTL14 (magenta) and a satellite glial cell marker (GFAP) (green) or macrophage marker (IBA1) (green) in the rat DRG 10 days after treatment with saline or PCX. PCX, paclitaxel; SAL, saline (n = 10 /group, Student's t-test) Scale bar: 50 µm. Data are shown as the mean ± SEM.

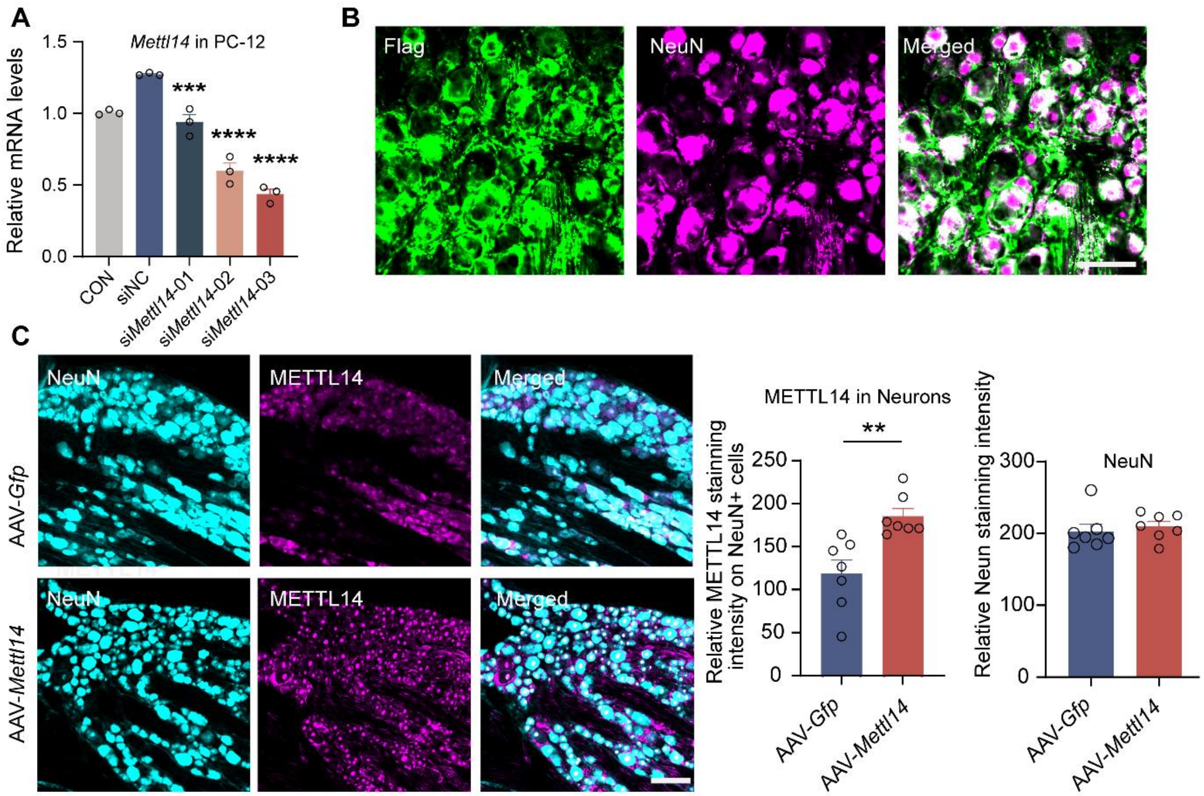

Supplemental Figure 2. In vitro siRNA sequence screening and in vivo virus efficiency validation. (A) *Mettl14* mRNA expression in PC-12 cells transfected with siRNA targeting *Mettl14* (siRNA1,2,3), scrambled siRNA and the control (one-way ANOVA followed by Dunnett's post hoc test). (**B**) Flag labels showing high colocalization with neurons (NeuN-positive cells) in rat DRG. Scale bar: 50 µm. (**C**) Double-labeled immunofluorescent images showing the colocalization and alteration of METTL14 in neurons (marked by NeuN). Bar plots showing the increased relative METTL14 intensity in NeuN positive cells after AAV application (n = 7 /group, Student's t-test). Scale bar: 100 µm. Data are shown as the mean ± SEM. ** $P < 0.01$, *** $P < 0.001$, **** $P < 0.0001$.

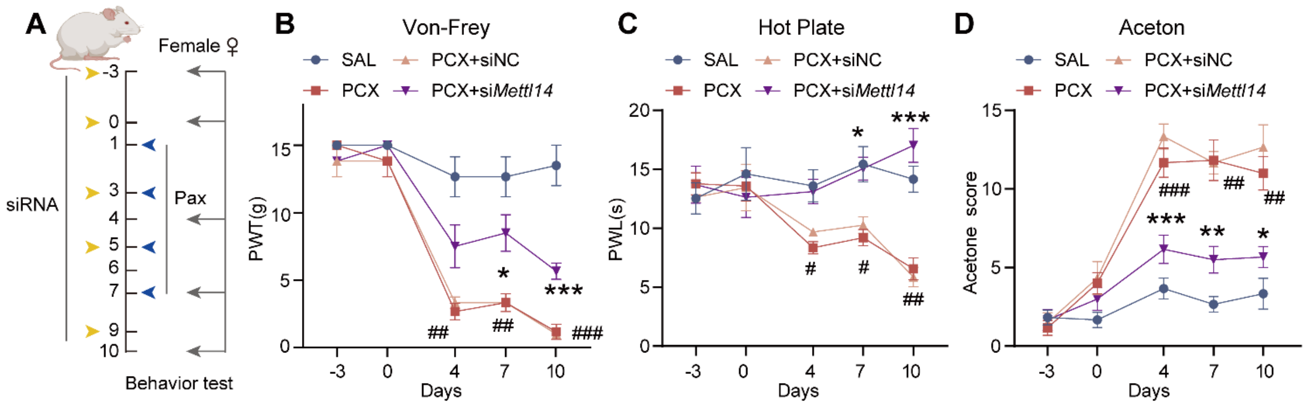

Supplemental Figure 3. Sex-dependent effects and therapeutic role of METTL14 in rats. (**A**) Illustration of the experimental design in female rats. (**B-D**) Mechanical allodynia, thermal hyperalgesia and cold allodynia of saline- or PCX-treated female rats after si*Mettl14* or scrambled siRNA treatment measured by the von Frey test (B), hot plate test (C) and acetone test (D), respectively (* PCX compared to SAL, # PCX+si*Mettl14* compared to PCX+siNC, at least 6 rats/group, two-way ANOVA followed by Tukey's post hoc test). * $P < 0.05$, ** $P < 0.01$, *** $P < 0.001$. # $P < 0.05$, ## $P < 0.01$, ### $P < 0.001$.

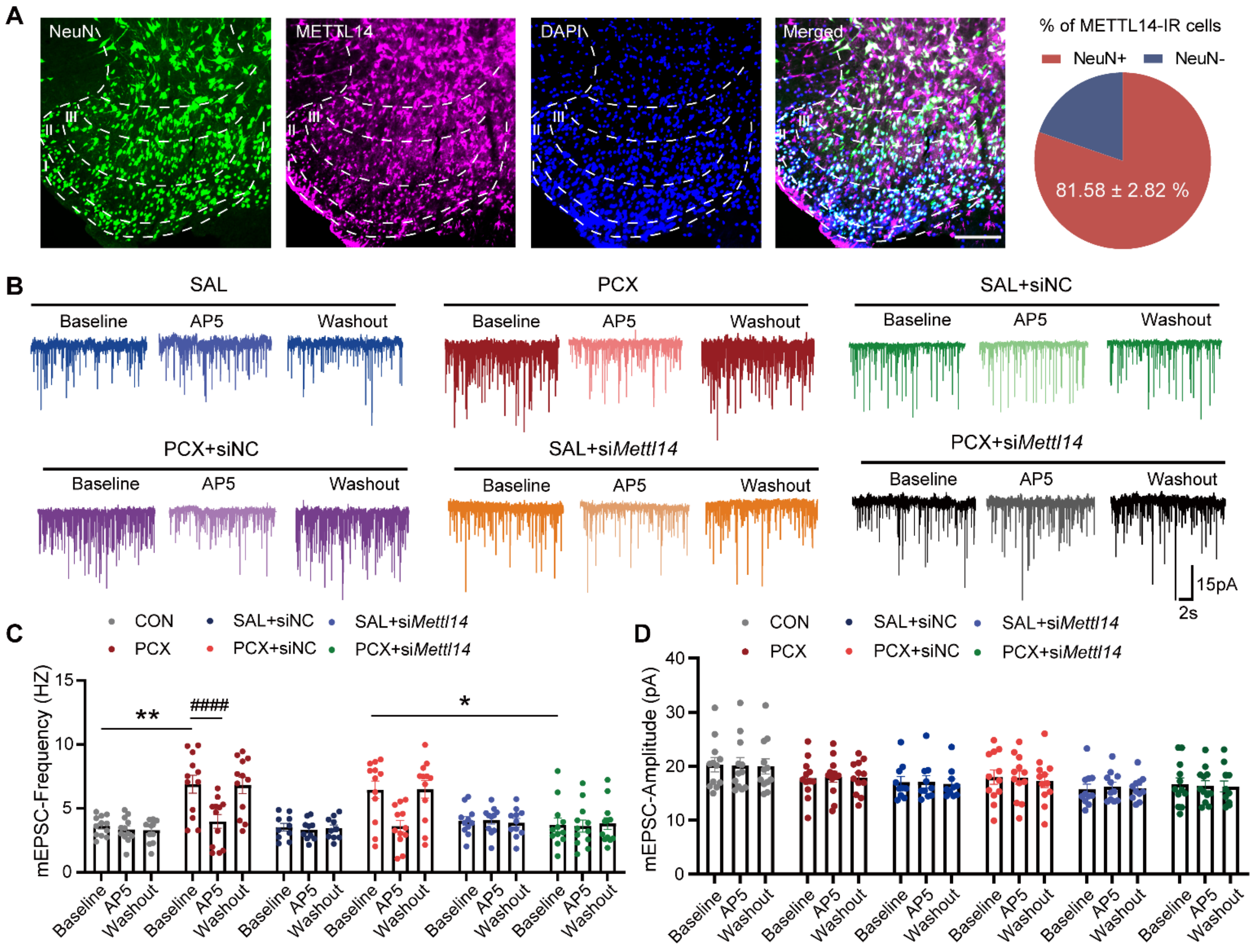

Supplemental Figure 4. METTL14 expression in spinal dorsal horns and in mediating NMDAR hyperactivity. (**A**) Representative double immunofluorescence staining and quantification showing high colocalization of METTL14 with NeuN in spinal dorsal horns. Pie charts illustrating the proportion of NeuN-positive cells in METTL14 immunoreactivity (METTL14-IR) cells in spinal dorsal horns (n= 12 slices from 6 rats) Scale bar: 50 µm. (**B**) Representative recording traces show the effect of bath application of AP5 on the frequency and amplitude of mEPSCs of lamina II neurons from saline- or PCX-treated rats after si*Mettl14* or scrambled siRNA. (**C, D**) Bar plots show the changes in the frequency and amplitude of mEPSCs of lamina II neurons during baseline control and bath application of AP5 in saline- or PCX-treated rats after siRNA-*Mettl14* or scrambled siRNA treatment (two-way ANOVA followed by Tukey's post hoc test). For whole-cell patch-clamp recording, at least 3 rats and 10 neurons/group were used. Data are shown as the mean ± SEM. * P < 0.05. ** P < 0. 01 (compared with baseline between groups). ### P < 0.0001 (compared with the respective baseline).

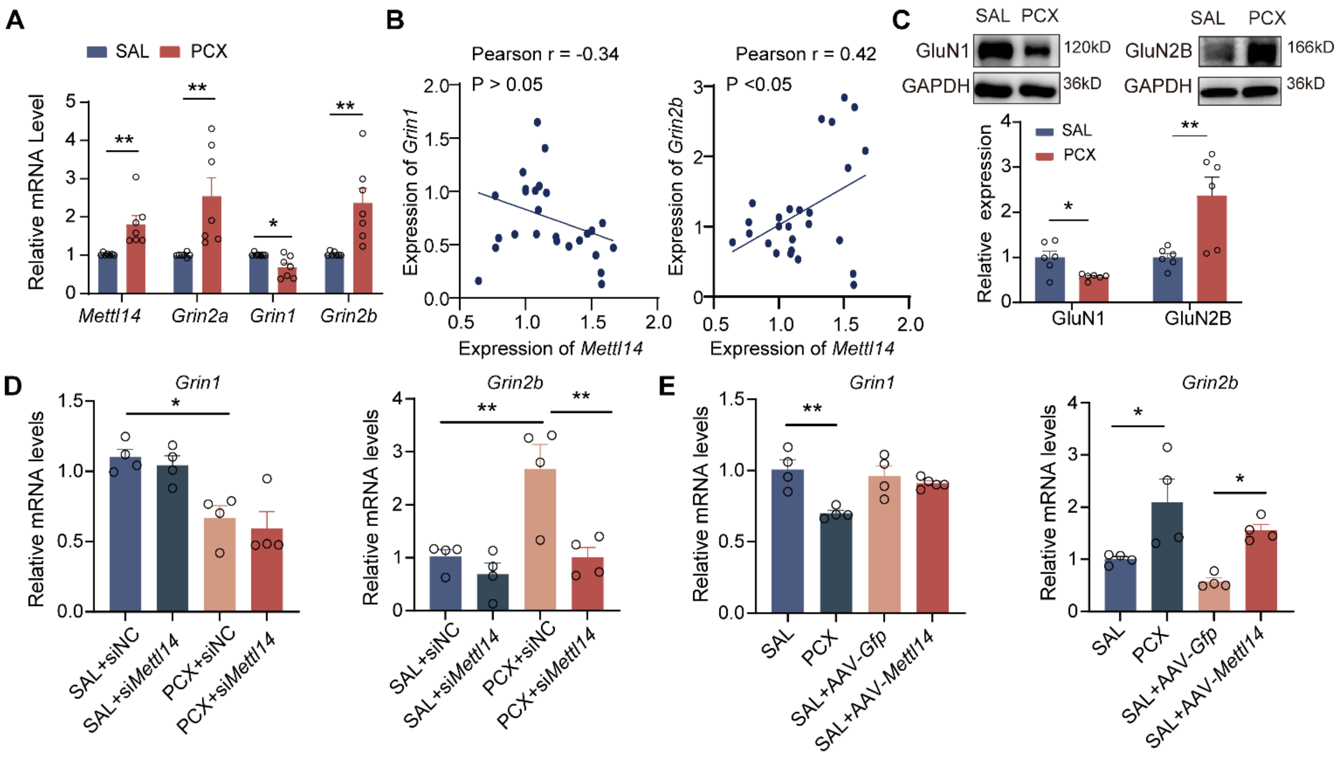

Supplemental Figure 5. *Grin2a*, but not *Grin1* or *Grin2b*, in the DRG, is a downstream target of METTL14 after paclitaxel treatment. (**A**) *Mettl14*, *Grin2a*, *Grin1*, and *Grin2b* mRNA expression in the bilateral L4-6 DRGs in saline- or PCX-pretreated rats (n = 7 rats/group, Student's t-test). **(B)** Correlations between *Grin2b*, *Grin1*, and *Mettl14* mRNA expression were calculated using Pearson's correlation**.** (**C**) GluN1 and GluN2B protein expression in the bilateral L4-6 DRGs in saline- or PCX-pretreated rats (n = 6 rats/group, Student's t-test). (**D**) *Grin1* and *Grin2b* expression in the bilateral L4-6 DRGs in saline- or PCX-pretreated rats after siRNA-*Mettl14* or scrambled siRNA intrathecal injection. (n = 4 rats/group, one-way ANOVA followed by Tukey's post hoc test). (**E**) *Grin1* and *Grin2b* expression in the bilateral L4-6 DRGs in saline- or PCX-pretreated rats after AAV-*Mettl14* or AAV-*Gfp* intrathecal injection (n = 4 or 5 rats/group, one-way ANOVA followed by Tukey's post hoc test). Data are shown as the mean ± SEM. * P < 0.05, ** P < 0.01.

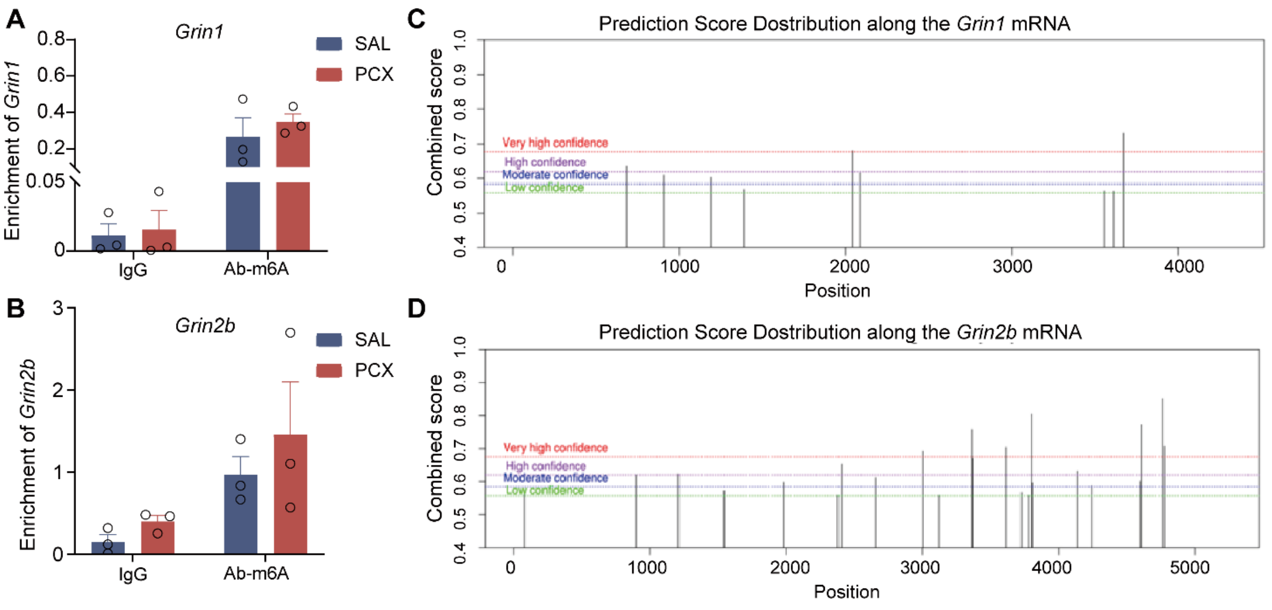

Supplemental Figure 6. ***Grin1*** **and** ***Grin2b*** **are not downstream targets of METTL14-mediated m6A modification after paclitaxel treatment. (A, B**) RIP-qPCR results for enriched *Grin1* and *Grin2b* mRNA in vivo using an m6A antibody compared to IgG control (two-way ANOVA followed by Sidak's post hoc test). (**C, D**) Prediction of the m6A methylation sites in *Grin1* and *Grin2b* mRNA by SRAMP program.

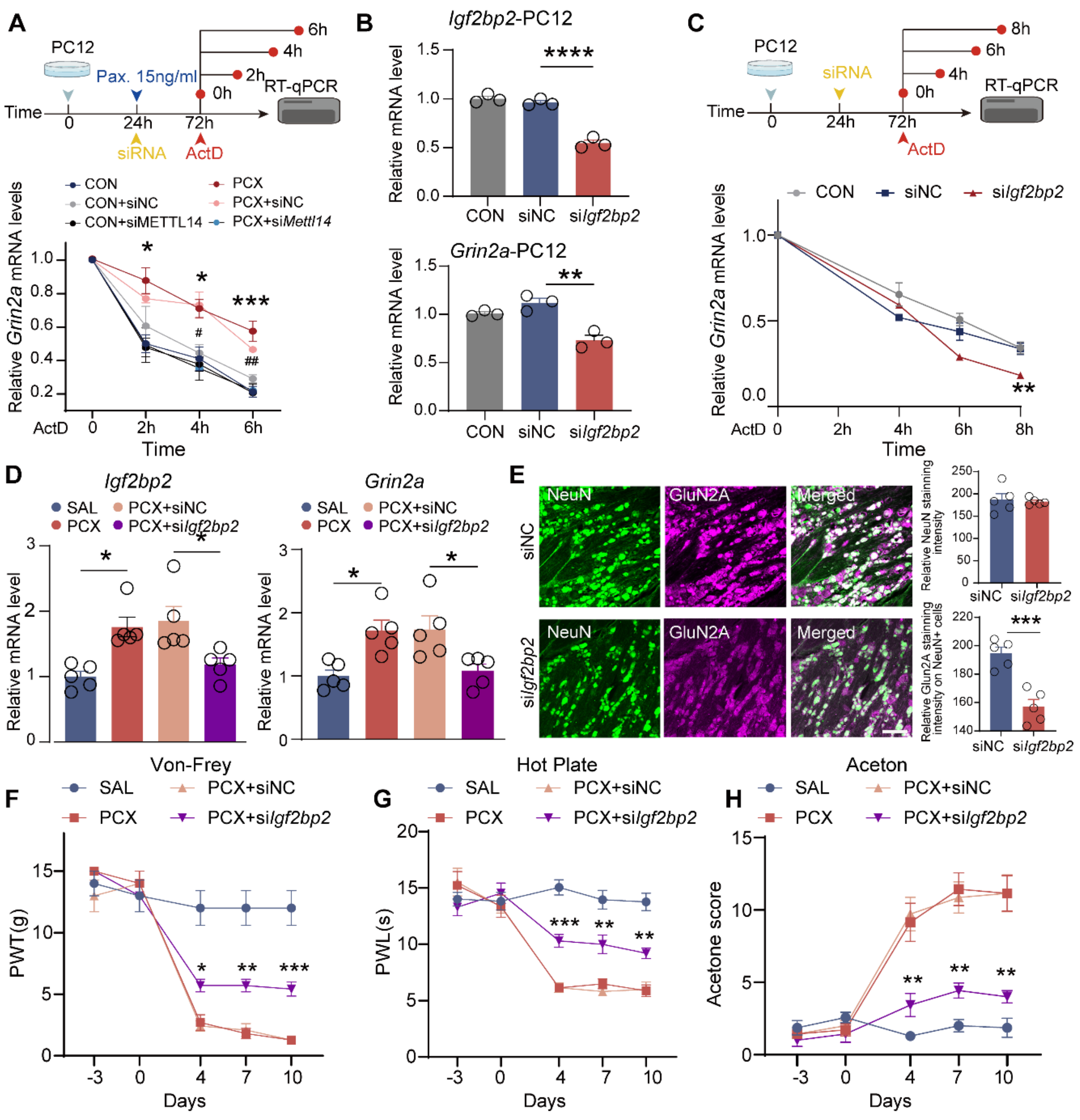

Supplemental Figure 7. IGF2BP2 contributes to GluN2A expression in DRG neurons and regulates pain behaviors. (A) Upper lane: Schematic diagram of RNA stability assay in PC-12. Lower lane: RNA stability in ActD-incubated PC-12 cells transfected with siRNA-*Mettl14* or scrambled siRNA under saline- or PCX-pretreated conditions (one-way ANOVA followed by Tukey's post hoc test at each time point). *P< 0.05 (PCX compared with SAL). # P < 0.05 (PCX+si*Mettl14* compared with PCX+siNC). (**B**) *Igf2bp2* and *Grin2a* mRNA expression in PC-12 after si*Igf2bp2* transfection (n = 3/group, one-way ANOVA followed by Tukey's post hoc test). (**C**) RNA stability curves plotted in ActD-incubated PC-12 cells transfected with siRNA-*Igf2bp2* or scrambled siRNA compared to the controls (two-way ANOVA followed by Tukey's post hoc test). (**D**) *Igf2bp2* and *Grin2a* mRNA expression in the bilateral L4-6 DRGs in saline- or PCX-pretreated rats after siRNA-*Igf2bp2* intrathecal injection (n = 5/group, one-way ANOVA followed by Tukey's post hoc test). (**E**) Double-labeled immunofluorescent images and quantification show the colocalization and alteration of GluN2A in neurons (marked by NeuN) after si*Igf2bp2* application (n = 5 rats/ group, Student's t-test). Scar bar = 50μm. (**F-H**)

Mechanical allodynia, thermal hyperalgesia and cold allodynia of saline- or PCX-treated rats after si*Mettl14* or scrambled siRNA treatment measured by the von Frey test (F), hot plate test (G) and aceton test (H), respectively (at least 6 rats/group, two-way ANOVA followed by Tukey's post hoc test). * P < 0.05. ** P < 0. 01. *** P < 0. 001 (PCX+si*Igf2bp2* compared with PCX+siNC rats).

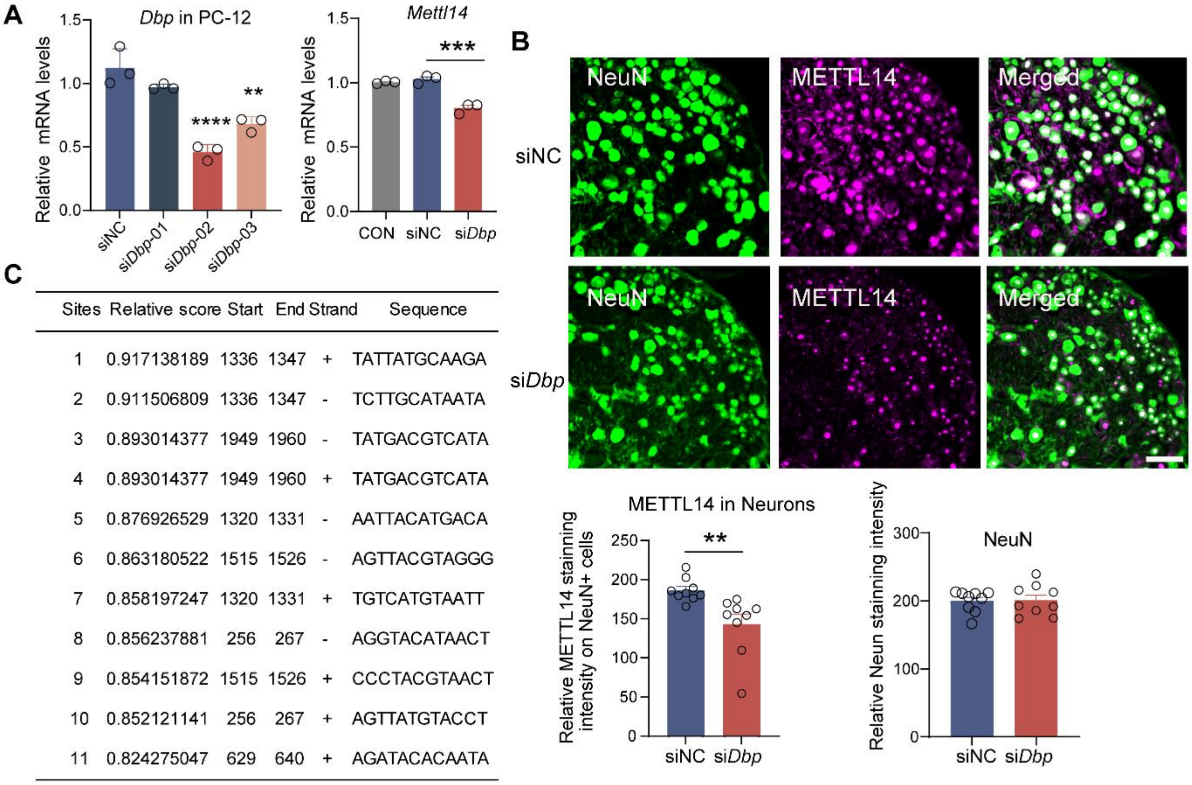

Sites	Relative score	Start	End	Strand	Sequence
1	0.917138189	1336	1347	+	TATTATGCAAGA
2	0.911506809	1336	1347	-	TCTTGCATAATA
3	0.893014377	1949	1960	-	TATGACGTCATA
4	0.893014377	1949	1960	+	TATGACGTCATA
5	0.876926529	1320	1331	-	AATTACATGACA
6	0.863180522	1515	1526	-	AGTTACGTAGGG
7	0.858197247	1320	1331	+	TGTCATGTAATT
8	0.856237881	256	267	-	AGGTACATAACT
9	0.854151872	1515	1526	+	CCCTACGTAACT
10	0.852121141	256	267	+	AGTTATGTACCT
11	0.824275047	629	640	+	AGATACACAATA

Supplemental Figure 8. DBP contributes to METTL14 increased expression in DRG neurons. (**A**) *Dbp* and *Mettl14* mRNA expression in PC-12 cells transfected with siRNA targeting *Dbp* (si*Dbp*1, 2, 3) and scrambled siRNA. (n = 3 /group, one-way ANOVA followed by Tukey's post hoc test). (B) Double-labeled immunofluorescent images and quantification showing the colocalization and downregulation of METTL14 in neurons (marked by NeuN) after si*Dbp* application. Bar plots show the relative METTL14 intensity in NeuN positive cells and NeuN intensity (n = 9 rats/ group, Student's t-test). Scar bar= 50 μm. (C) Predictive DBP binding sites in *Mettl14* promoter sequence by JASPAR (Relative score > 0.8). ** P < 0.01, *** P < 0.001, **** P < 0.0001.

Supplemental Tables

Supplemental Table 1. Characteristics of patients included.

ID	Gender	Age	BMI	Segment	Diagnosis	Chemotherapeutic agents
P1	Male	60	23.5	T8	Nasopharyngeal carcinoma	Not applicable
P2	Male	61	26.7	T12	Lung cancer	Not applicable
P3	Male	43	26.9	T7	Plasmacytoma	Not applicable
P4	Male	57	24.9	T4	Ureter carcinoma	Gemcitabine、 Carboplatin
P5	Female	63	22.2	T12	Plasmacytoma	Bortezomib、Vincristine、Doxorubicin
P6	Female	57	23.6	T8	Breast cancer	Epirubicin, Docetaxel

Supplemental Table 2. The Nucleotide Sequences of siRNA

Gene	Species	Sequence
METTL14	Rat	5‘- CCTTTGACATCAGAGAATT -3
IGF2BP2	Rat	5‘- GGAAGTGATCGTCAGAATT -3’
DBP	Rat	5‘- GAAACAGCAAGCCCAAAGA -3’

Supplemental Table 3. Specific Primer Sequences for RT-qPCR or RIP-PCR.

Gene	Sequence
Mettl14	F 5'-GCAGAAACCTACGCGTCCTA-3'
	R 5'-CACCACGGTCAGACTTGGAT-3'
Grin2a	F 5'-CGACCCCGGCAGCTTTGGAA-3'
	R 5'-GCGAGTGGGTCCGATTCTCTGC-3'
Igf2bp1	F 5'-AGGATCTCACGCTCTATAACCC-3'
	R 5'-CCACGTCATTCTCGTAAGCC-3'
Igf2bp2	F 5'-AAGTTATAGTGCCTCGTGACC-3'
	R 5'-ATGCCCGATAATTCTGACGAT-3'
Igf2bp3	F 5'-CCACCATTCGCAACATCACC-3'
	R 5'-GCCCCTGTATTCTCCTTACGAT-3'
Dbp	F 5'-GCGGTTAGTGGCGGCTT-3'
	R 5'-GCTGAGGCTTCAATTCCTCCT -3'
Grin1	F 5'-TGGCATCATCGGACTTCAG-3'
	R 5'-TCTGGTGGACATCTGGTATC-3'
Grin2b	F 5'-CCTGGAATGGCATGATCG-3'
	R 5'-AGCCACCGCAGAAACAAT-3'
Gapdh	F 5'-TGCCACTCAGAAGACTGTGG-3'
	R 5'-TTCAGCTCTGGGATGACCTT-3'
