# Supplementary material for: METTL14-mediated m6A epitranscriptomic modification contributes to chemotherapy-induced neuropathic pain by stabilizing GluN2A expression via IGF2BP2
Source: J Clin Invest. 2024 Feb 6;134(6):e174847. doi: 10.1172/JCI174847 (PMC10940092; doi:10.1172/JCI174847)
Supplement: Unedited blot and gel images [file jci-134-174847-s177.pdf]

Full unedited gel for Figures in

METTL14-mediated m6A epitranscriptomic modification contributes to chemotherapy-induced neuropathic pain by stabilizing GluN2A expression via IGF2BP2

Weicheng Lu[1,†], Xiaohua Yang[1,†], Weiqiang Zhong[1,†], Guojun Chen[1,†], Xinqi Guo[2], Qingqing Ye[1], Yixin Xu[1], Zhenhua Qi[1], Yaqi Ye[1], Jingyun Zhang[3], Yuge Wang[3], Xintong Wang[1], Shu Wang[1], Qiyue Zhao[2], Weian Zeng[1], Junting Huang[3*], Huijie Ma[2*], Jingdun Xie[1*]

[1] Department of Anesthesiology, State Key Laboratory of Oncology in South China, Sun Yat-sen University Cancer Center, Guangzhou, Guangdong 510060, China.

[2] Department of Physiology, Hebei Medical University, Shijiazhuang, Hebei, 050017, China.

[3] Department of Anatomy and Neurobiology, Zhongshan School of Medicine, Sun Yat-sen University, Guangzhou, Guangdong 510080, China

Full unedited gel for Figure1F

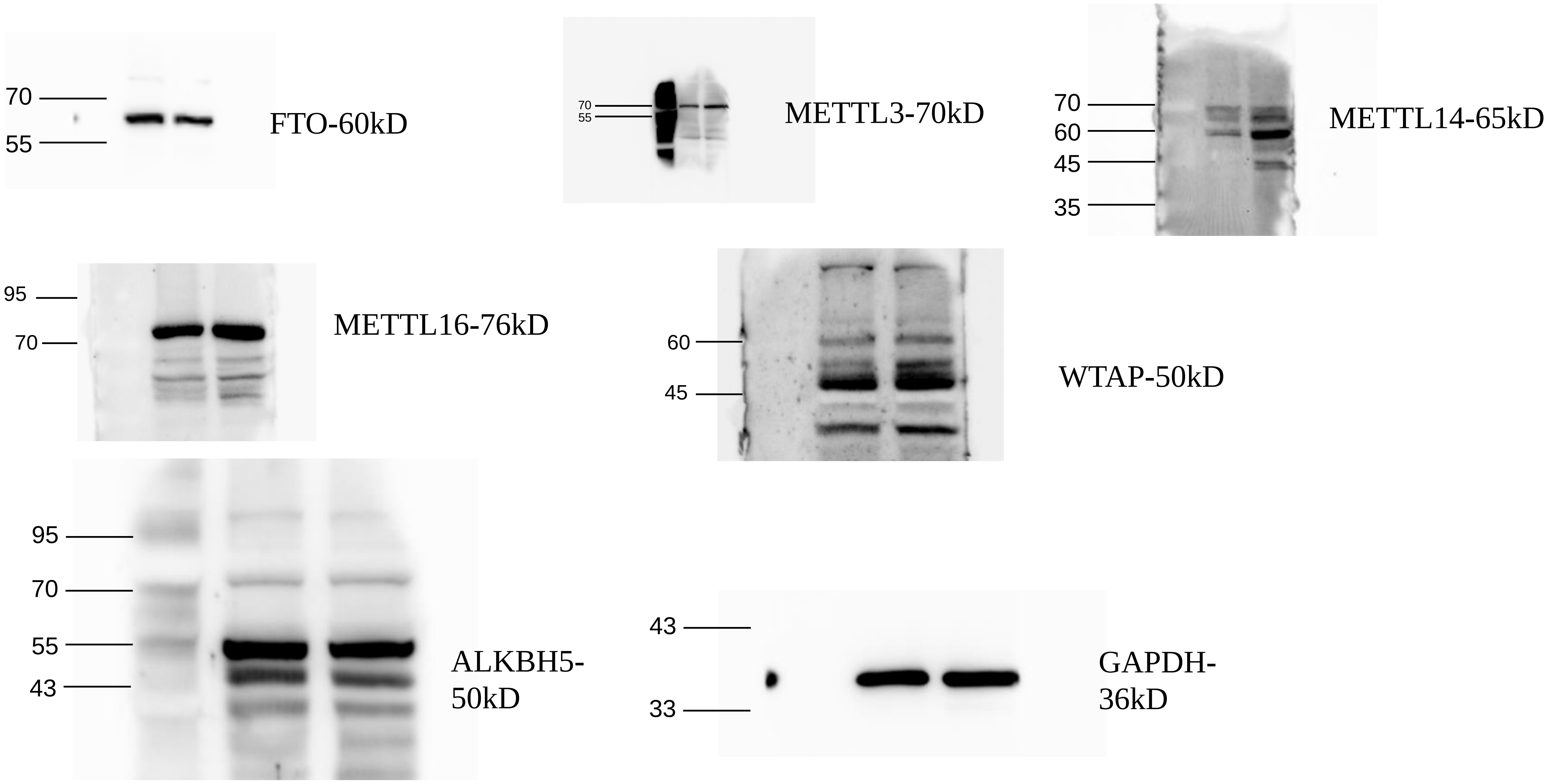

Full unedited gel for Figure 1H

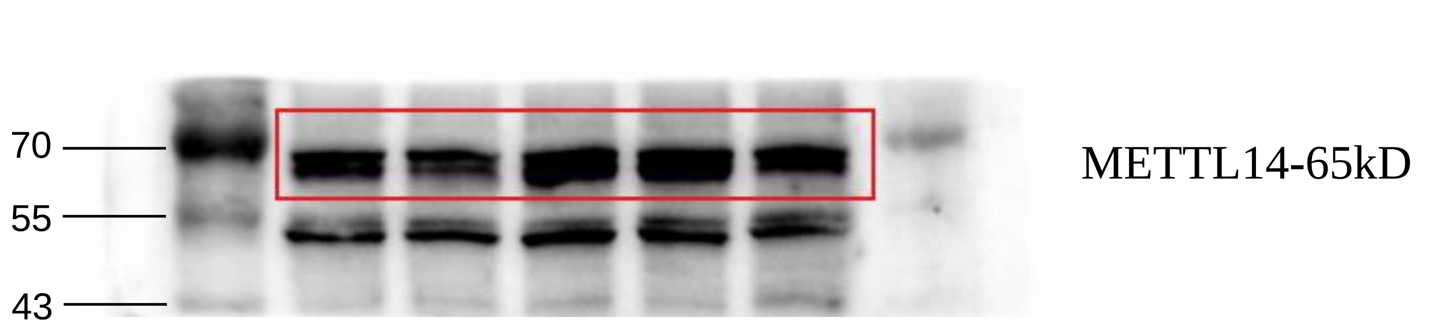

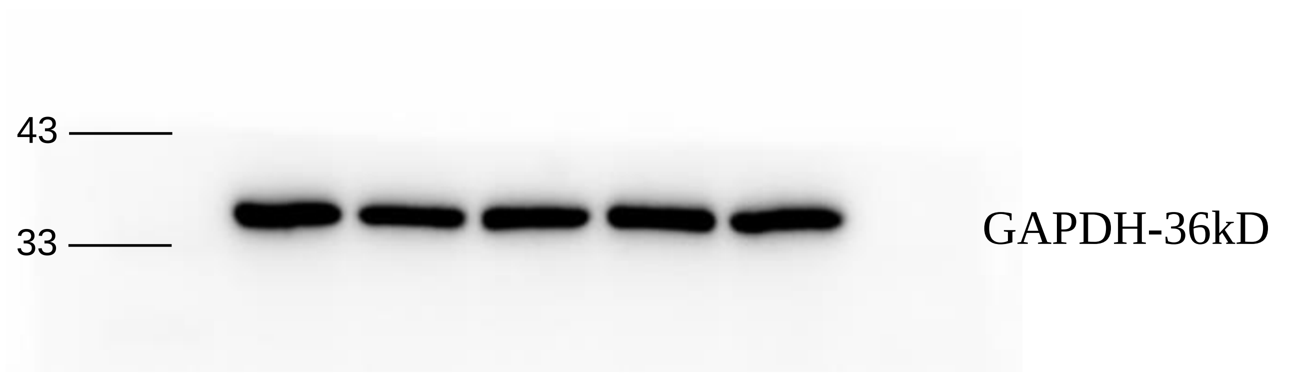

Full unedited gel for Figure 3B

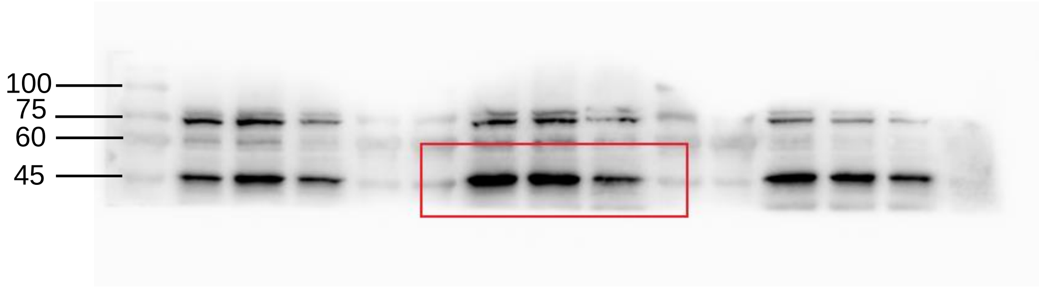

METTL14-65kD

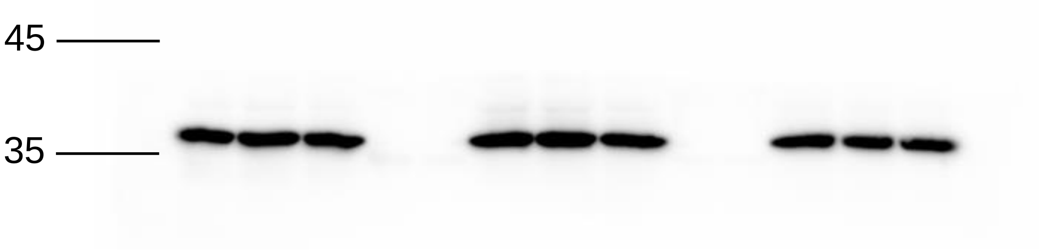

GAPDH-36kD

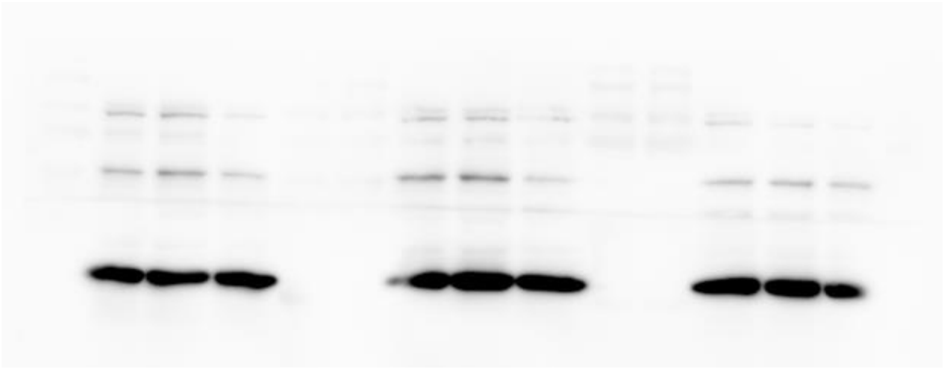

Merged

Full unedited gel for Figure 3E

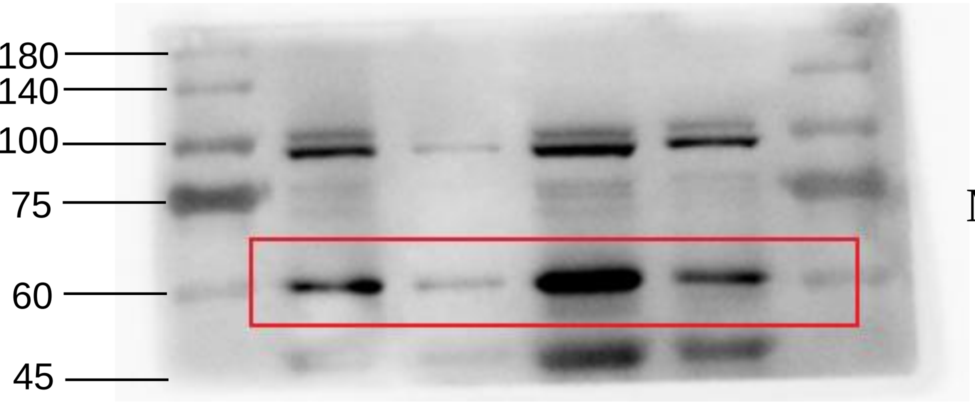

METTL14-65kD

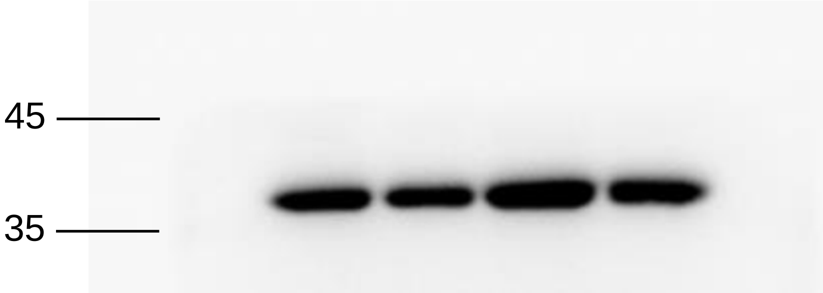

GAPDH-36kD

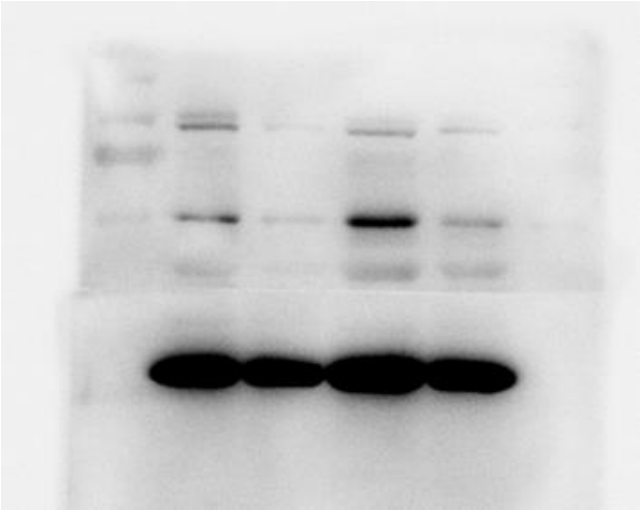

Merged

Full unedited gel for Figure 3H

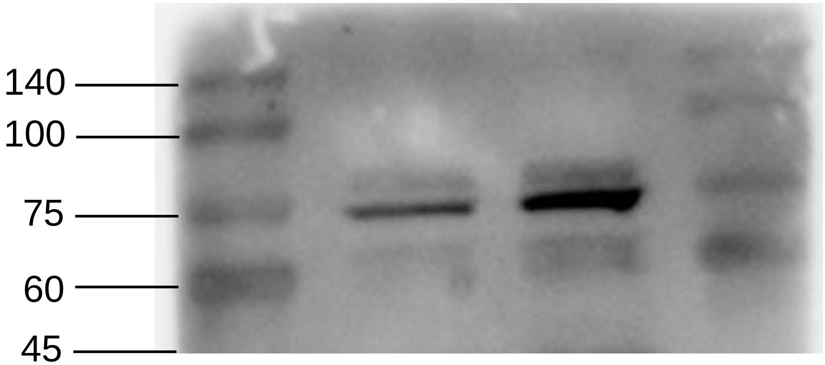

METTL14-65kD

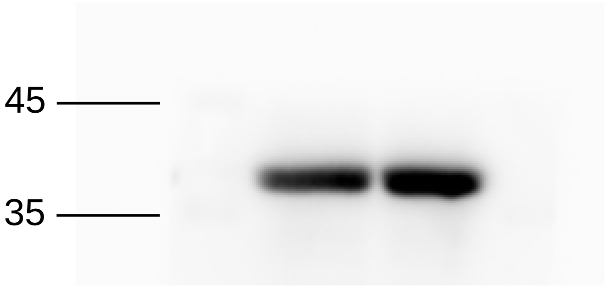

GAPDH-36kD

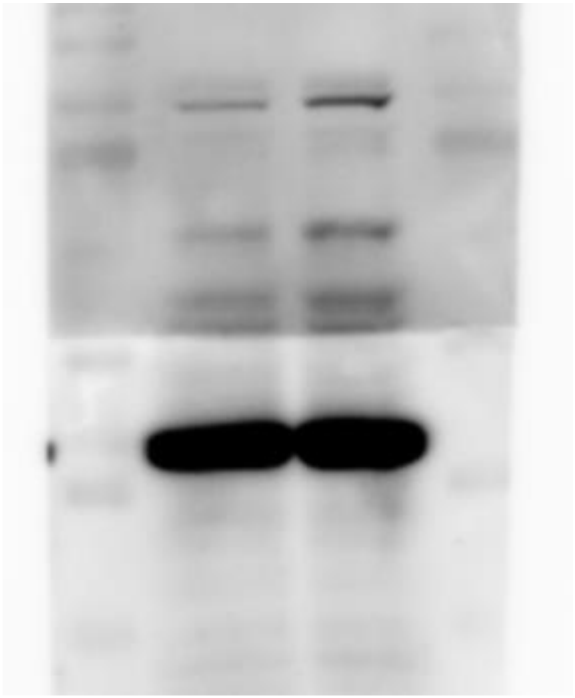

Merged

Full unedited gel for Figure 6B

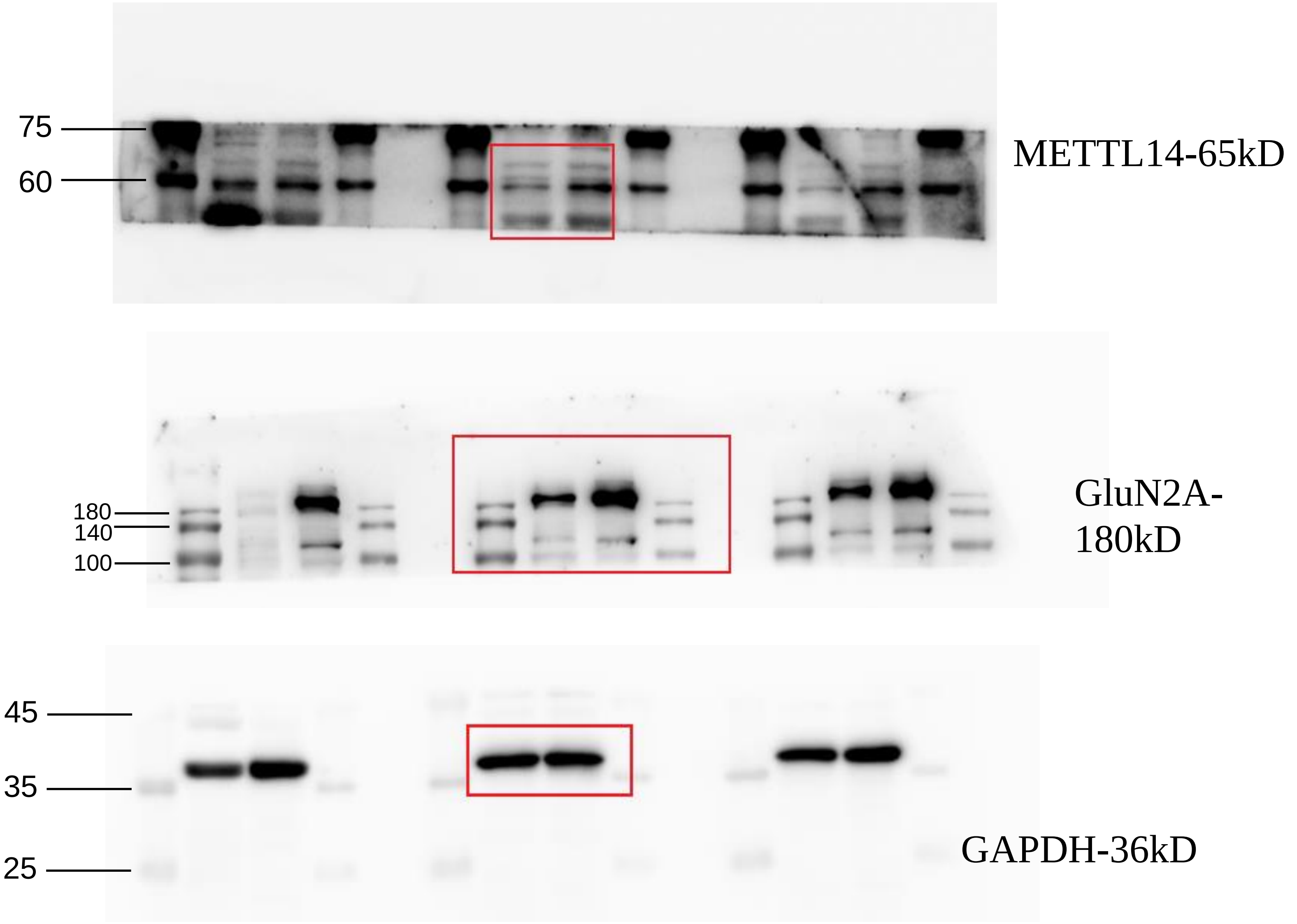

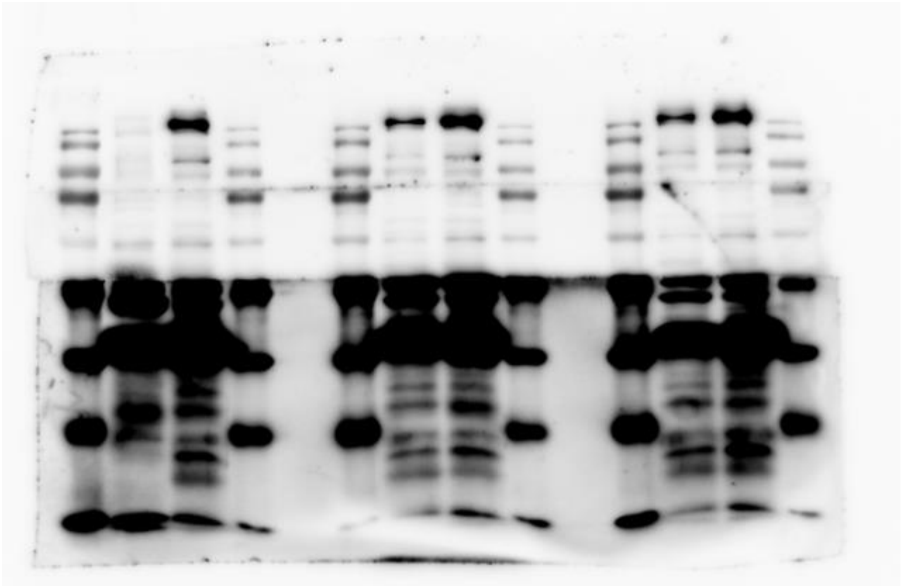

Merged

Full unedited gel for Figure 6D

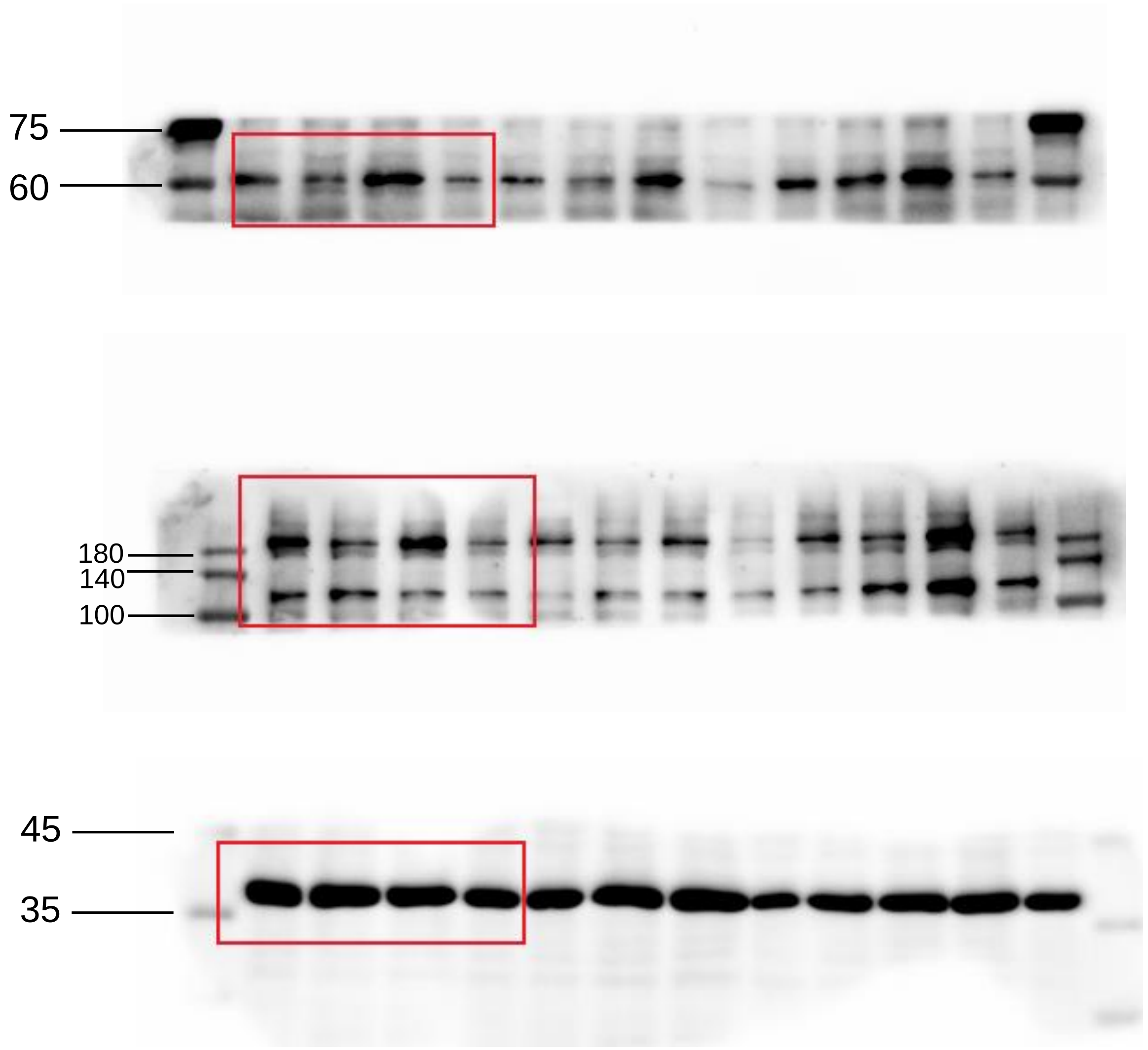

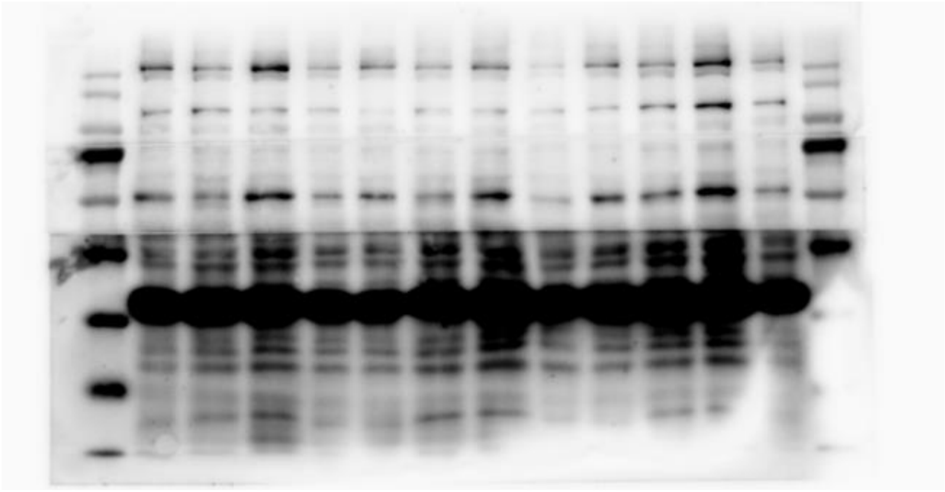

Merged

Full unedited gel for Figure 6F

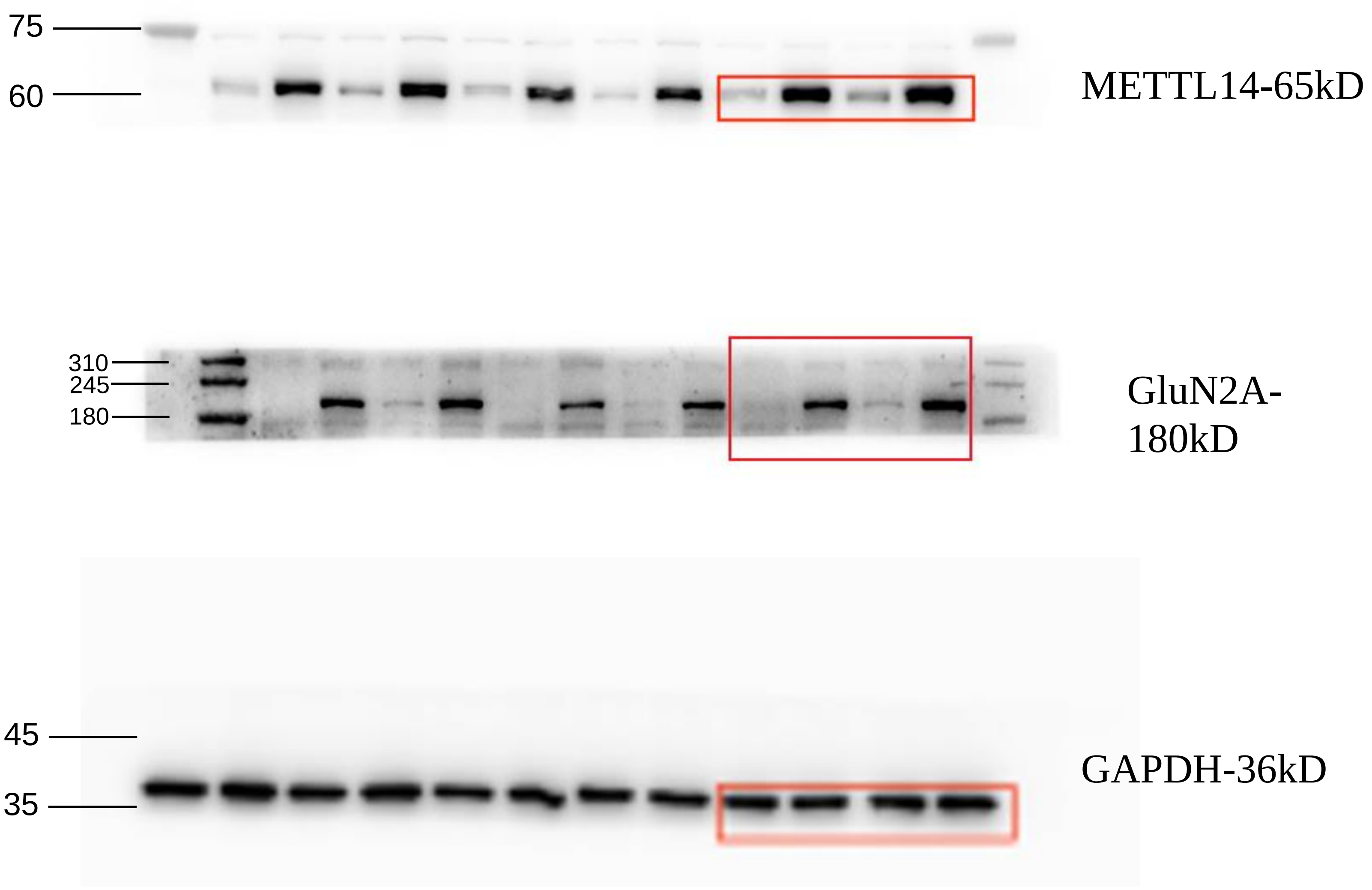

Full unedited gel for Figure 8F

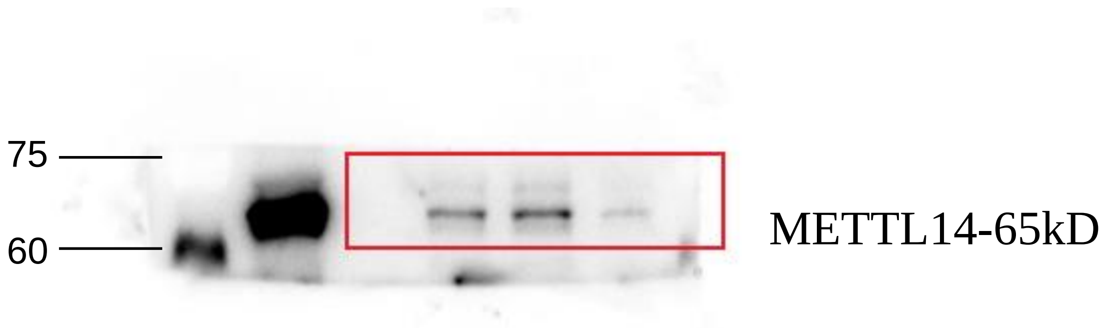

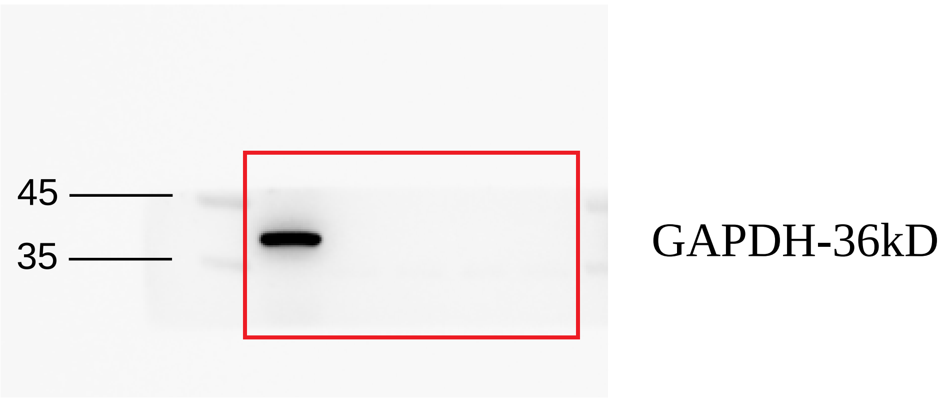

Full unedited gel for Figure 9F

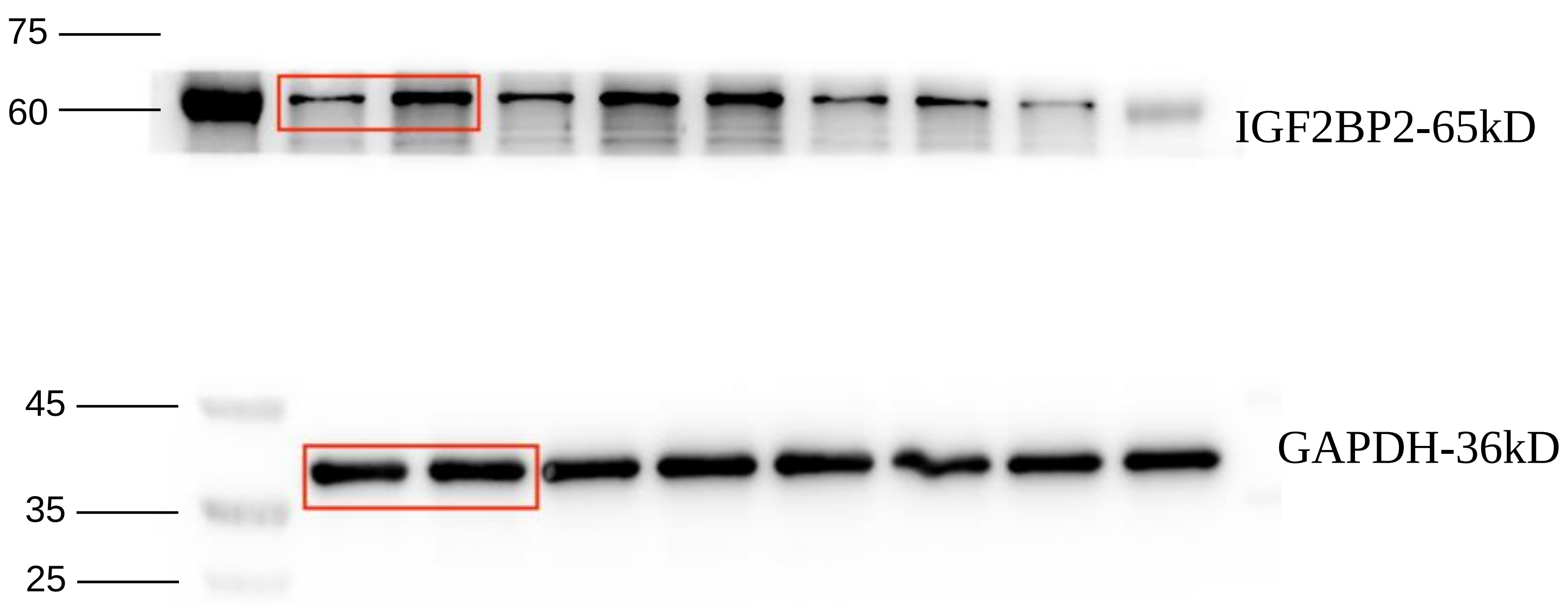

Full unedited gel for Figure 9G

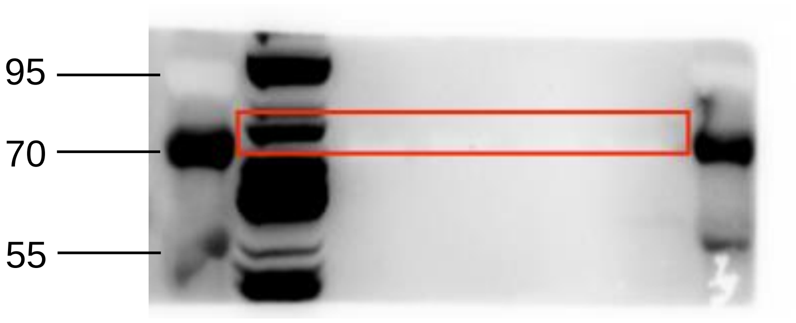

IGF2BP1-71kD

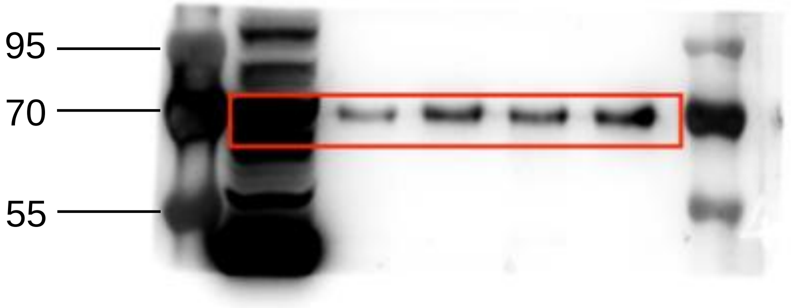

IGF2BP2-65kD

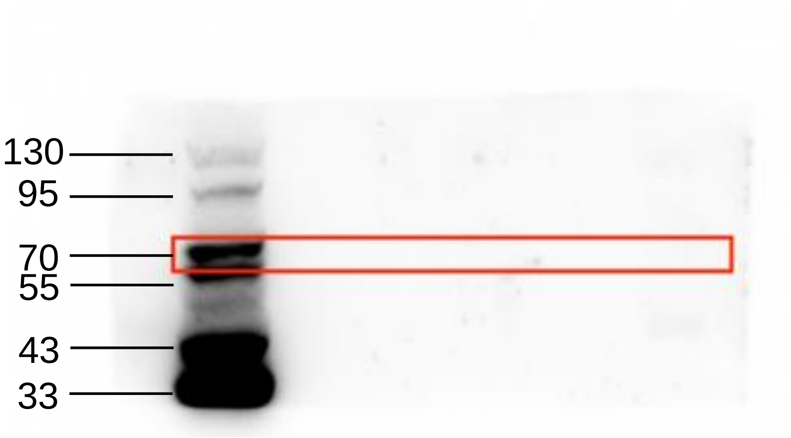

IGF2BP3-70kD

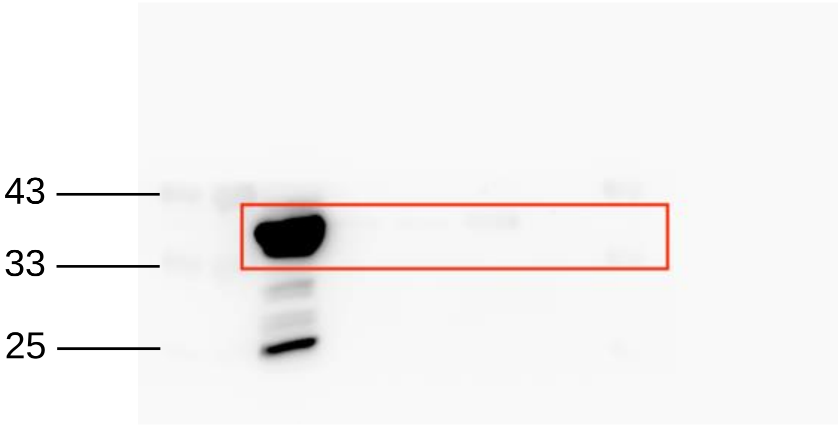

GAPDH-36kD

Full unedited gel for Figure 10C

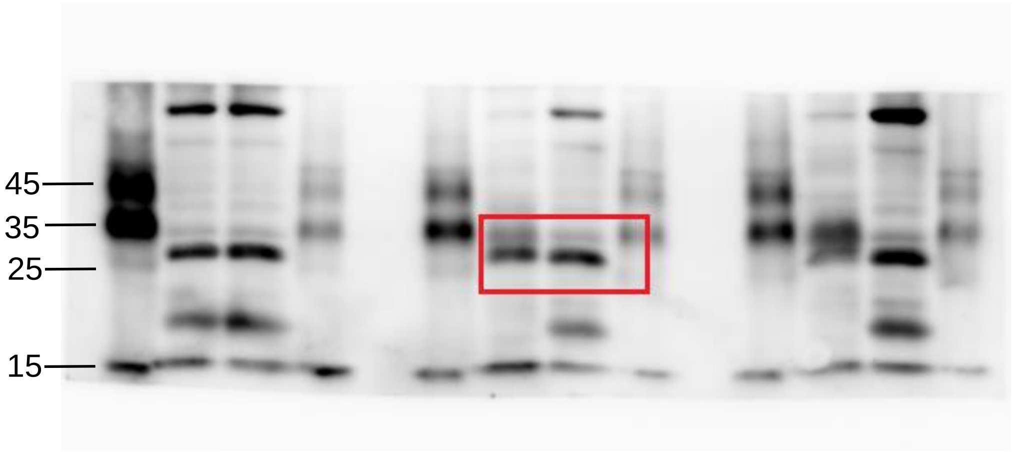

DBP-34kD

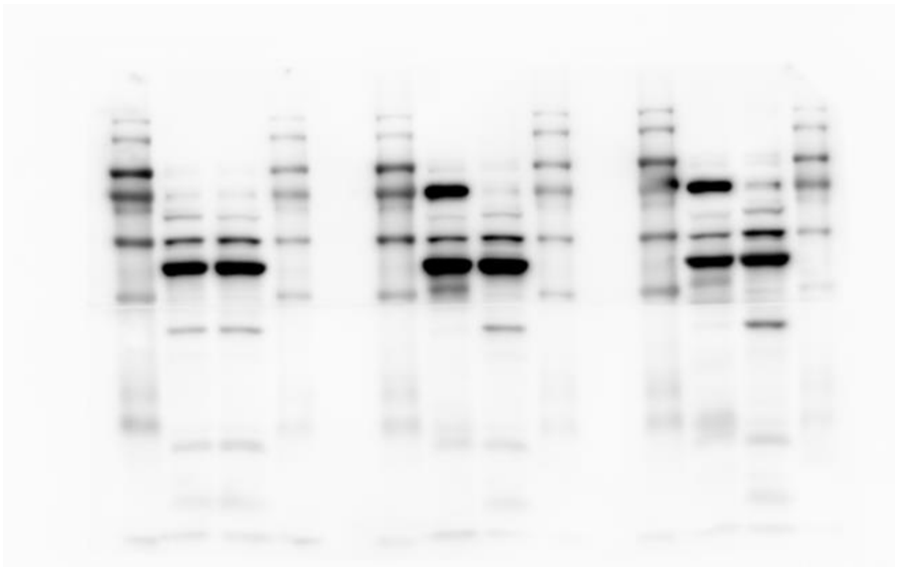

Merged

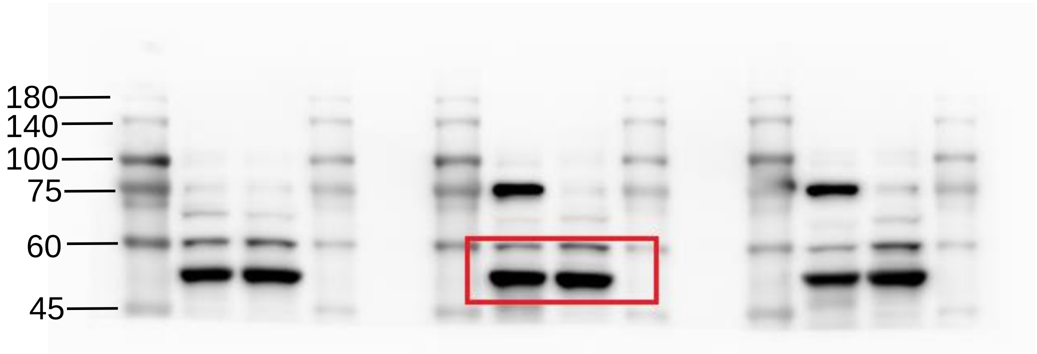

β-tubulin-50kD

Full unedited gel for Figure 10E

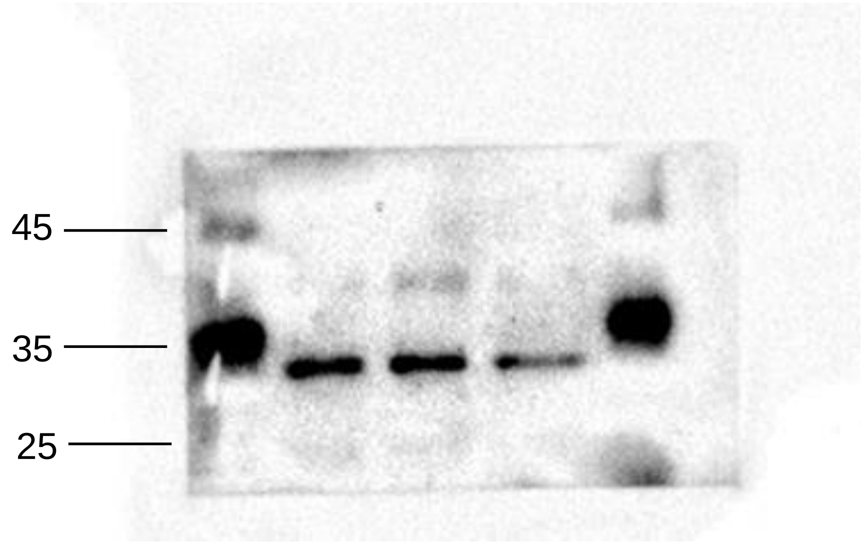

DBP-34kD

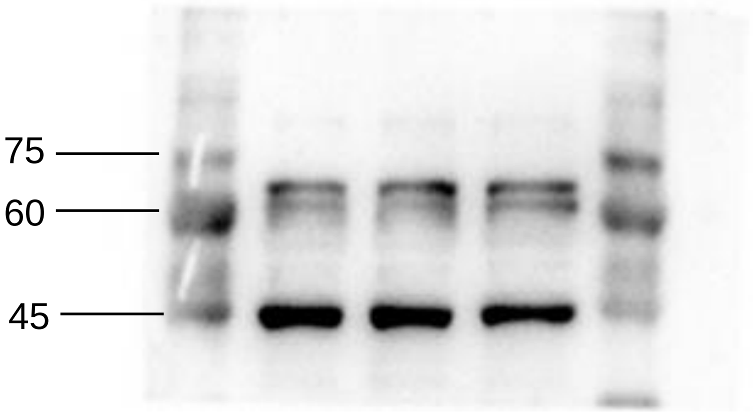

β-tubulin-50kD

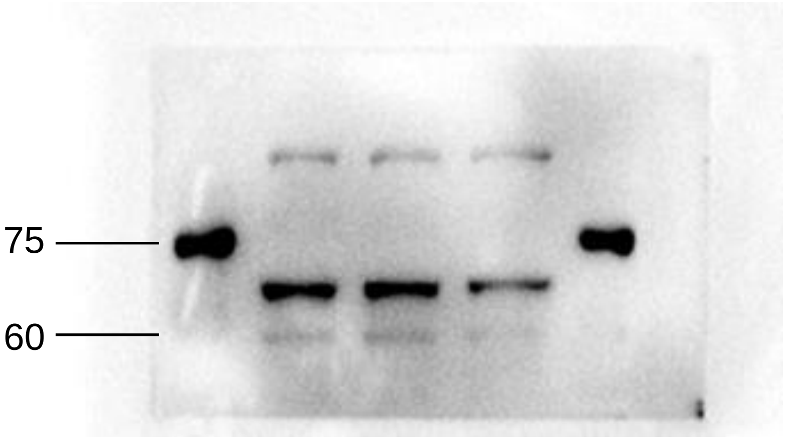

METTL14-65kD

Full unedited gel for Figure S5C

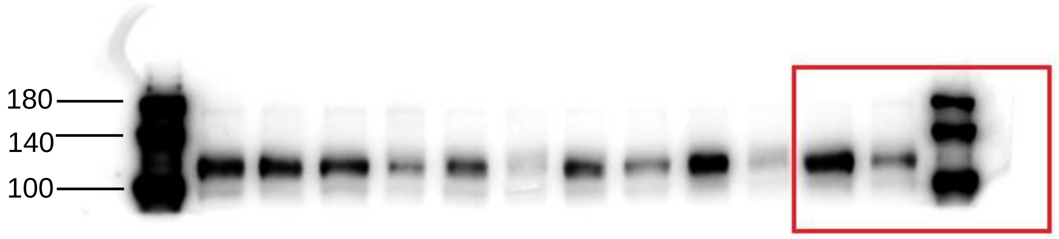

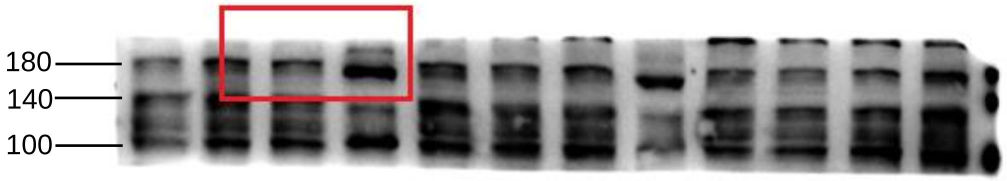

GluN2B-166kD

GluN1-120kD

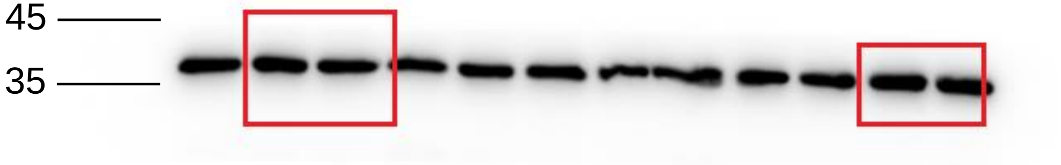

GAPDH for GluN2B

36kD

GAPDH for GluN1

36kD
